# Inferring transcriptional logic from multiple dynamic experiments

**DOI:** 10.1093/bioinformatics/btx407

**Published:** 2017-06-28

**Authors:** Giorgos Minas, Dafyd J Jenkins, David A Rand, Bärbel Finkenstädt

**Affiliations:** 1 Mathematics Institute, University of Warwick, Coventry, CV4 7AL, UK; 2 Zeeman Institute, Systems Biology and Infectious Disease Epidemiology Research, University of Warwick, Coventry, CV4 7AL, UK; 3 Department of Statistics, University of Warwick, Coventry, CV4 7AL, UK

## Abstract

**Motivation:**

The availability of more data of dynamic gene expression under multiple experimental conditions provides new information that makes the key goal of identifying not only the transcriptional regulators of a gene but also the underlying logical structure attainable.

**Results:**

We propose a novel method for inferring transcriptional regulation using a simple, yet biologically interpretable, model to find the logic by which a set of candidate genes and their associated transcription factors (TFs) regulate the transcriptional process of a gene of interest. Our dynamic model links the mRNA transcription rate of the target gene to the activation states of the TFs assuming that these interactions are consistent across multiple experiments and over time. A trans-dimensional Markov Chain Monte Carlo (MCMC) algorithm is used to efficiently sample the regulatory logic under different combinations of parents and rank the estimated models by their posterior probabilities. We demonstrate and compare our methodology with other methods using simulation examples and apply it to a study of transcriptional regulation of selected target genes of Arabidopsis Thaliana from microarray time series data obtained under multiple biotic stresses. We show that our method is able to detect complex regulatory interactions that are consistent under multiple experimental conditions.

**Availability and implementation:**

Programs are written in MATLAB and Statistics Toolbox Release 2016b, The MathWorks, Inc., Natick, Massachusetts, United States and are available on GitHub https://github.com/giorgosminas/TRS and at http://www2.warwick.ac.uk/fac/sci/systemsbiology/research/software.

**Supplementary information:**

Supplementary data are available at *Bioinformatics* online.

## 1 Introduction

Elucidating the structure of gene regulation from biological data is a key task of systems biology with applications spanning across biology and biomedicine (Levine *et al.*, 2014; Marbach *et al.*, 2010). Rapid development of a range of high-throughput technologies is giving rise to the generation of genome-wide time course mRNA measurements, while public repositories permit the wide distribution and sharing of these data (Hecker *et al.*, 2009). Due to the advancement of experimental protocols and techniques facilitating perturbations at both the cellular and whole organism level, genome-wide data can be collected under a range of conditions. Hence, researchers now have access to an unparalleled level of information regarding gene expression and network dynamics under multiple experimental conditions (Goda *et al.*, 2008; Hickman *et al.*, 2013; Kilian *et al.*, 2007; Ou-Yang *et al.*, 2017). Moreover, high-throughput technologies such as yeast-one-hybrid (Y1H, Ouwerkerk and Meijer, 2001), ChIP-chip and ChIP-seq, DNase-seq and ATAC-seq (Meyer and Liu, 2014) can identify protein-DNA interactions and thus putative transcription factors (TFs) for target genes.

On the methodological side, a substantial literature of mathematical, statistical and computational approaches often termed *reverse-engineering* or *network inference methods* focus on inferring interactions between a large number of genes from high-throughput expression profiles (see Kiani *et al.*, 2016; Hecker *et al.*, 2009). They employ a number of different methodological approaches including co-expression and clustering algorithms (Faith *et al.*, 2007; Margolin *et al.*, 2006), Boolean logic models (Bornholdt, 2008; Han *et al.*, 2014), Bayesian networks (Thorne and Stumpf, 2012; Yu *et al.*, 2004) and differential equation models (Madar *et al.*, 2010; Titsias *et al.*, 2012; Yip *et al.*, 2010). Differential equation models can potentially describe mechanistic relations between target genes and their TFs but they often use large numbers of parameters and face model identifiability issues (Hecker *et al.*, 2009). Boolean logic models can describe complex regulations of target genes including AND, OR, XOR co-regulations but require a preprocessing step of discretization of the continuous scale expression profiles to typically binary (ON/OFF) levels.

When dealing with datasets from multiple experimental conditions, most of the currently available methods derive one network structure for each experimental condition or even replicate of the same experiment. The exceptions are the approach in Penfold *et al.* (2012) that combines a hierarchical structure of the network with a set of global ‘average regulators’ extracted in addition to local regulators and the approach in Wang *et al.* (2006), Weber *et al.* (2013) and Ou-Yang *et al.* (2017) that attempts to extract consistent networks across conditions. We follow the latter approach.

We introduce the transcriptional regulation switch (TRS) model that employs ordinary differential equations (ODEs) linking the transcription of the target gene to the observed activation states of a set of potential regulators by means of a piecewise linear transcription rate function which jumps to a different value when at least one regulator in the set changes its activation (ON/OFF) state. This form of transcription function is a simple and flexible model that can also be seen as an approximation of the commonly used S-shaped (e.g. Hill type) functions (Alon, 2014; Klipp *et al.*, 2016). The number and identity of the regulators as well as the logic of the regulation are unknown and need to be estimated. Each of the observed activation states of the set of regulators is associated with a single value of the transcription rate of the regulated gene across experimental conditions and over time. This constrains the parameterization and empowers the method to identify consistent regulatory connections between a regulated gene and its TFs that hold under multiple conditions as in Wang *et al.* (2006), Ou-Yang *et al.* (2017) and Weber *et al.* (2013).

The latter studies describe the target transcription rate using linear or non-linear regression models (also used in Madar *et al.*, 2010 and Yip *et al.*, 2010) that can at most capture additive TF effects. This modelling approach substantially limits the regulatory interactions that can be derived. For example, interactive co-regulations such as the simple regulation of a target gene with two TFs A and B where B suppresses the activation caused by A cannot be captured by those models as we discuss in more detail later. Such an interaction is experimentally observed e.g. the homeotic gene fushi tarazu (ftz) related to the development of *Drosophila melanogaster* (Latchman, 2005). Auto-regulation cannot be captured either as it cannot be distinguished from mRNA degradation.

We will show that TRS can detect these types of interactive regulations while Boolean type regulations such as AND, OR and XOR activations and repressions (see Bornholdt, 2008; Han *et al.*, 2014) can be derived without the need of arbitrary data discretization. Despite the complexity of these regulation models and because our approach limits the estimation of transcription rates to only those values that are associated with activation states that are observed in the given dataset, the parameter identifiability issues that complex mechanistic models often face (see Hecker *et al.*, 2009) are avoided.

Bayesian statistical methodology is used for the inference on model parameters including the number and identity of the most likely regulators, their action as activators, repressors and/or co-regulators and the logic of this regulation. Our approach for inference is implemented through a trans-dimensional reversible jump Markov chain Monte Carlo (RJMCMC) algorithm (Green, 1995; Jenkins *et al.*, 2013). An advantage of this approach is that it returns all plausible regulation models along with their posterior probabilities. Therefore, the search and selection between possible sets of regulators is done during the MCMC run and no additional post-processing computationally expensive scoring or selection steps are needed.

Two simulation examples are used to illustrate the approach. An artificial repressed activation network is considered along with a published regulation model related to the flowering time of *A. thaliana* (Leal Valentim *et al.*, 2015). Our results are compared with the outcomes of GRNInfer tool in Wang *et al.* (2006); Ou-Yang *et al.* (2017). The methodology is then applied to study the transcriptional regulation of two chosen target genes of *A. thaliana* under multiple biotic stresses.

## 2 Methods

### 2.1 TRS model

Consider the regulation of the mRNA transcription of a target gene by an unknown set $\Phi =\{\phi_{1},\phi_{2},\ldots ,\phi_{\nu }\}$ of TFs. As in the temporal transcriptional switch model of Jenkins *et al.* (2013) we assume that the joint mRNA expression of the target gene over a population of cells may be described by a piece-wise linear ODE where mRNA transcription is decoupled from mRNA degradation and allowed to change or *switch* between different states. The mRNA expression, *M*(*t*), of the target gene is hence described by

(1) $$\frac{dM}{dt}=\tau (t)-\delta M(t),\;t\in [0,L]$$

where *δ* is the mRNA degradation rate and τ(t) the transcription rate (TR) function. This is piece-wise constant with jumps at (unknown) time-points *s_i_*, i=1,2,…,m, where the transcription rate moves from some value, $\tau_{i-1}$, to a higher (lower) value *τ_i_*, which we will refer to as activation (deactivation).

Extending the univariate approach of Jenkins *et al.* (2013), the transcriptional switches in our model occur when the TF level, $P_{\phi }(t),\;t\in (0,L)$, of a regulator ϕ∈ Φ crosses a threshold, $\rho_{\phi }$, to change between active and inactive states. Furthermore, the TRs, *τ_i_*, i=0,1,…,m are linked to the activation states of the regulators in Φ such that each state of the activation function $\alpha (t)=(\alpha_{1}(t),\ldots ,\alpha_{\nu }(t))$, where, for j=1,…,ν, t∈[0,L],

$$\alpha_{j}(t)=\{\begin{array}{c} 0,\;P_{\phi_{j}}(t)<\rho_{\phi_{j}}\;(\phi_{j}\;\text{is}\;\text{inactive}),\\ 1,\;P_{\phi_{j}}(t)\geq \rho_{\phi_{j}}\;(\phi_{j}\;\text{is}\;\text{active}), \end{array}$$

is associated with a single value of the TR of the target gene across experimental conditions and over time. If $a_{1},a_{2},\ldots ,a_{q}$ are the observed states of α(t) for *t* in [0,L], then the TR function in (1) can be written as $\tau (t)=\sum_{i}^{q}\tau_{a_{i}}I_{a_{i}},$ where $I_{a_{i}}$ the indicator function $I_{a_{i}}=I(\alpha (t)=a_{i})$.

**Fig. 1.**
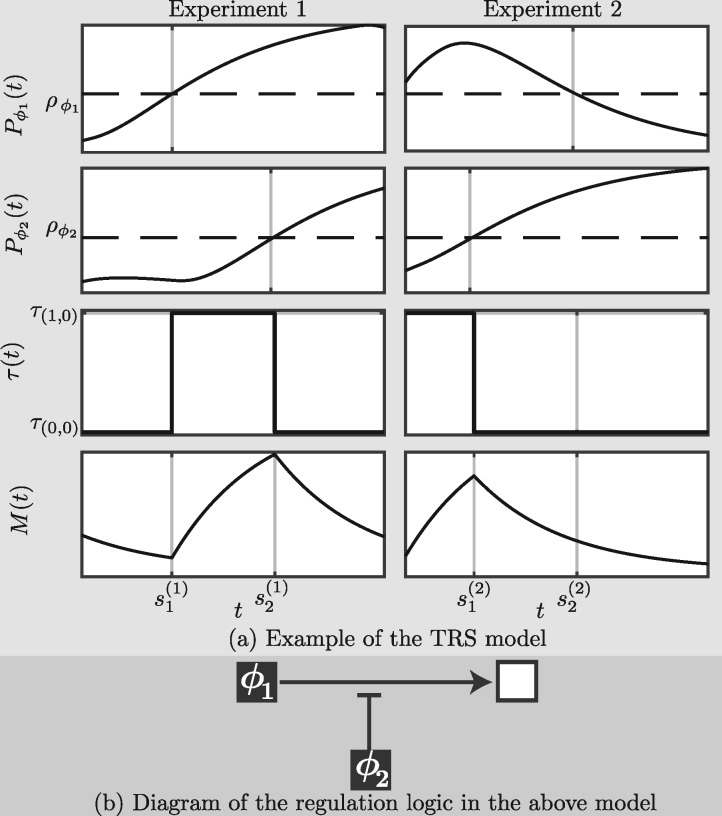
Example TRS model. The two top panels of Figure (**a**) display the profiles (solid lines), $P_{\phi_{1}}$ and $P_{\phi_{2}}$, of regulators, $\phi_{1}$ and $\phi_{2}$, and their threshold levels (dashed lines), $\rho_{\phi_{1}}$ and $\rho_{\phi_{2}}$, in two experiments (left and right). The 3rd and 4th rows respectively show the TR function τ(t) and the mRNA expression profile *M*(*t*) of the target under the TRS model in each experiment. The activation of $\phi_{1}$ at time $s_{1}^{\left(1\right)}$ of the experiment 1 produces the ‘on’ switch of the TR $\tau_{\left(0,0\right)}\to \tau_{\left(1,0\right)}$. This activation is repressed in experiment 2 where the $\tau_{\left(0,1\right)}\to \tau_{\left(1,1\right)}$ switch at $s_{2}^{\left(2\right)}$ does not change the TR ($\tau_{\left(0,1\right)}=\tau_{\left(1,1\right)}$). The activation of $\phi_{2}$ at $s_{2}^{\left(1\right)}$ and $s_{1}^{\left(2\right)}$ produced the ‘off’ switch $\tau_{\left(1,0\right)}\to \tau_{\left(1,1\right)}$. This suggests that $\phi_{2}$ inhibits the activation of the target by $\phi_{1}$. The regulatory states $a_{1}=(1,0)$ and $a_{2}=(1,1)$ are observed in multiple time intervals constraining constraining τ(t) to be equal to $\tau_{\left(1,0\right)}$ for $t\in [s_{1}^{\left(1\right)},s_{2}^{\left(1\right)}]\cup [0,s_{1}^{\left(2\right)}]$ and $\tau_{\left(1,1\right)}$ for $t\in [s_{2}^{\left(1\right)},L^{\left(1\right)}]\cup [s_{1}^{\left(2\right)},s_{2}^{\left(2\right)}]$. Figure (**b**) displays a diagram of the logic of the regulation model with → indicating activation and ⊣ suppression

Figure 1 displays a simple example of the TRS model with two regulators, namely an activator and a repressor of this activation, observed under two experimental conditions. Note that, as we discuss in Sect. 1 of SI, the additive linear and non-linear models of transcription, such as in Wang *et al.* (2006), cannot deduce such regulatory interactions.

**Fig. 2.**
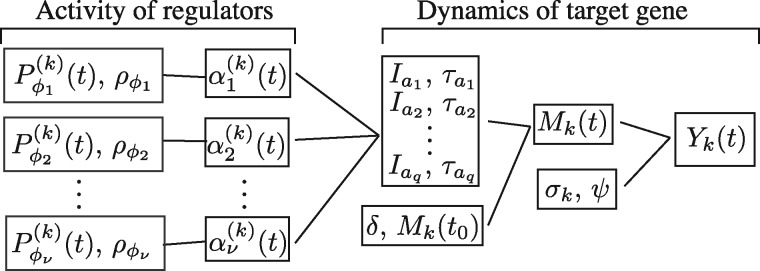
Graphical representation of the parameters of the TRS model. The profiles of the TFs, $P_{\phi_{j}}^{\left(k\right)}$, and their thresholds, $\rho_{\phi_{j}}$, define their activation functions, $\alpha_{j}^{\left(k\right)}(t)$, and these in turn the observed activation states $a_{1},a_{2},\ldots ,a_{q}$ and the associated time-intervals, $I_{a_{i}}$ and TRs, $\tau_{a_{i}}$, which along with the initial conditions and the degradation rate provide the model to be fitted to the mRNA expression of the target

For given threshold levels, $\rho_{\Phi }=(\rho_{\phi_{1}},\ldots ,\rho_{\phi_{\nu }})$, of the regulators in Φ, the time intervals $I_{a_{k}}\subseteq [0,L],\;I_{a_{k}}\neq \emptyset$ can be obtained and the general solution of the model in (1) can be written as

(2) $$\begin{array}{c} M(t)=M(0)\;e^{-\delta t}+\tau_{a_{1}}e^{-\delta t}\int_{[0,t]\cap I_{a_{1}}}e^{\delta u}du\\ \;\;+\cdots +\tau_{a_{q}}e^{-\delta t}\int_{[0,t]\cap I_{a_{q}}}e^{\delta u}du, \end{array}$$

which implies that, for fixed $\rho_{\Phi }$ and degradation rate *δ*, the ODE solution of our model has the form of a linear regression with coefficients *M*(0), $\tau_{a_{1}},\ldots ,\tau_{a_{q}}$. The number of regressors depends on the activation function of the regulators. Our methodology described below can accommodate a variable number of regulators and experiments and provides a probabilistic classification of the posterior credibility of the corresponding logics of interaction (see also Fig. 2).

### 2.2 Bayesian inference

The aim is to identify the subset Φ of the set of all candidate regulators, $F=\{f_{1},f_{2},\ldots ,f_{N}\}$, that explains the observed expression of the target gene and to provide a description of the estimated regulatory associations.

Let $y=\left(y_{k}(t_{i})\right)$, be the observed mRNA expression of a target gene observed at time points $t_{1},t_{2},\ldots ,t_{n_{k}}$ for experiments k=1,…,K. A natural probabilistic assumption is that the observed time series are normally distributed with mean $M_{k}(t)$, equal to the ODE solution path in (1), and standard deviation $\sigma_{k}(t)=\sigma_{k}v_{k}(t)$. Here $v_{k}(t)={\left(w_{k}(t)\right)}^{-\psi_{k}}$ where $w_{k}(t)$ is a fixed time-dependent function and $\psi_{k}\in [0,1]$ is an unknown parameter (Gelman *et al.*, 2004).

The resulting log-likelihood function for the parameter set $\Theta =\{\Phi ,\rho_{\Phi },\delta ,M_{k}(t_{0}),\tau_{k}(t),t\in [0,L_{k}],\sigma_{k},\psi_{k},k=1,2,\ldots ,K\}$ is

(3) $$\text{log}\;L(\Theta ;y)=-\frac{1}{2}\sum_{k=1}^{K}\sum_{i=1}^{n_{k}}\;\text{log}\;(2\pi \sigma_{k}^{2}(t_{i}))+\frac{{\left(y_{k}(t_{i})-M_{k}(t_{i})\right)}^{2}}{\sigma_{k}^{2}(t_{i})}.$$

We note that different modeling assumptions, such as, for example the use of a different distribution of the measurement error, can be accommodated through other appropriate formulations of the likelihood function (see for example Strimmer, 2003).

To address the trans-dimensional nature of our model, we developed a reversible jump Markov chain Monte Carlo (RJMCMC) algorithm based on Green (1995) that allows for the Bayesian inference algorithm to move between models with a different numbers of regulators. Suppose that at a given iteration of the chain the value of the parameter vector is Θ. The next value, Θ′, is then derived as follows:

1. Draw and perform one of the following moves with probabilities *π_M_*, *π_S_*, *π_B_* and *π_D_*, respectively, where $\pi_{M}+\pi_{S}+\pi_{B}+\pi_{D}=1$.

M Move a threshold: randomly draw ϕ∈ Φ and replace $\rho_{\phi }$ with $\rho \prime_{\phi }∼\pi (\rho \prime_{\phi }|\rho_{\phi })$;

S Swap a regulator: randomly draw ϕ∈Φ and f′∈F∖ Φ and set Φ′=Φ∖{ϕ}∪{f′} and $\rho_{f\prime }∼\pi (\rho_{f\prime })$;

B Add a regulator (Birth): randomly draw f′∈F∖ Φ and set Φ′=Φ∪{f′} and $\rho_{f\prime }∼\pi (\rho_{f\prime })$;

D Remove a regulator (Death): randomly draw ϕ∈Φ and set Φ′=Φ∖ϕ.

2. Compute the likelihood L(Θ′;y) by the following steps:
 a. Use the updated regulator set and threshold levels to derive the observed activation states $a\prime_{1},\ldots ,a\prime_{q\prime }$ and the associated time intervals $I_{a\prime_{j}},\;j=1,\ldots ,q\prime$.
 b. Use least squares to estimate the transcription function $\tau \prime_{k}(\cdot )$ and initial conditions $M\prime_{k}(t_{0})$ as in Eq. (2).
 c. Compute the updated mRNA expression, $M\prime_{k}(t)$, of the target in each experiment, k=1,…,K using Eq. (2) and the likelihood L(Θ′;y) using Eq. (3).
3. Compute the acceptance ratio
  $$a(\Theta ,\Theta \prime )=\frac{\pi (\Theta \prime )}{\pi (\Theta )}\times \frac{L(\Theta \prime ;y)}{L(\Theta ;y)}\times \frac{\pi (\Theta |\Theta \prime )}{\pi (\Theta \prime |\Theta )},$$

where π(Θ) denotes the prior probability of Θ and π(Θ′|Θ) denotes the probability of the move Θ→ Θ′. Set Θ=Θ′ with probability min⁡{1,a(Θ,Θ′)}.

4. Propose δ′∼π(δ′|δ) and accept with probability min⁡{1,a(δ,δ′)} where
  $$a(\delta ,\delta \prime )=\frac{\pi (\delta \prime )}{\pi (\delta )}\times \frac{L(\delta \prime ;y)}{L(\delta ;y)}\times \frac{\pi (\delta |\delta \prime )}{\pi (\delta \prime |\delta )}$$
5. Draw $\sigma \prime_{k}$ and $\psi \prime_{k}$ from their full-conditional posterior distributions $\pi (\sigma \prime_{k}|y,\Theta \setminus \{\sigma_{k}\})$ and $\pi (\psi \prime_{k}|y,\Theta \setminus \{\psi \prime_{k}\})$*k* = 1,…,*K*.

Steps 4 and 5 are standard Metropolis Gaussian random-walk and Gibbs steps, respectively, while the moves in step 1, which sample the regulatory associations, constitute trans-dimensional jumps. The first two moves in step 1, i.e. moving threshold (M) and swapping regulator (S), keep the same number of regulators, while the last two moves, adding (B) and removing (D) a regulator, change the number of regulators by 1.

#### 2.2.1 Proposal distributions

Following Green (1995), we set the move probabilities in step 1 as

$$\pi_{B}(\nu )=c\min\left\{1,\frac{\pi (\nu +1)}{\pi (\nu )}\right\},\pi_{D}(\nu )=c\min\left\{1,\frac{\pi (\nu )}{\pi (\nu +1)}\right\},$$

where *c* is a constant set as large as possible subject to $\pi_{B}(\nu )+\pi_{D}(\nu )\leq \pi_{BD}\in (0,1)$ for all numbers of regulators $\nu =1,2,\ldots ,\nu_{max}\leq N$. The latter ensures that $\pi_{B}(\nu )$ and $\pi_{D}(\nu )$ satisfy the balance equation $\pi (\nu )\pi_{B}(\nu )=\pi (\nu +1)\pi_{D}(\nu +1)$, for all $\nu <\nu_{max}$, while they are set as large as possible subject to their sum never exceeding a boundary *π_BD_* set to control the number of attempted trans-dimensional moves during MCMC sampling. The probabilities of (M) and (S), respectively, are chosen as $\pi_{M}(\nu )=\tilde{\pi }(1-\pi_{B}(\nu )-\pi_{D}(\nu ))$ and $\pi_{S}(\nu )=(1-\tilde{\pi })(1-\pi_{B}(\nu )-\pi_{D}(\nu ))$ with $\tilde{\pi }\in (0,1)$ controlling how we split the probability of model moves in the same-dimension between regulator swaps and threshold moves.

A truncated normal distribution is used for the proposal probability of the regulator threshold level $\pi (\rho \prime_{\phi }|\rho_{\phi })$ in move (M) with mean equal to the current value $\rho_{\phi }$, variance $\sigma_{\rho }^{2}$ tuned to control the magnitude of the jumps and truncation bounds restricting the jumps within the range, $R_{\phi }$, of the profile of ϕ across experiments. The truncation ensures that no regulator in Φ is redundant. A uniform distribution on the profile range $R_{\phi }$ for the unconditional proposal probability $\pi (\rho \prime_{\phi })$ is used for the newly sampled TFs in moves S and B.

To derive the initial values $M_{k}(t_{0})$ and transcription rate functions $\tau_{k}(t),\;k=1,2,\ldots ,K$ in step 2, we follow Denison *et al.* (1998) and Jenkins *et al.* (2013) and use least squares estimation on the linear model in Eq. (2) that substantially increases computational speed over a full Bayesian regression estimation while the differences in results, at least for our application, are indistinguishable. More specifically, here we apply the parametric weighted least squares method (see Gelman *et al.*, 2004) with weights $w_{k}(t)$ set equal to the inverse of a smoothing spline kernel estimate ${\hat{M}}_{k}(t)$ of the target mRNA expression. The parameter $\psi_{k}\in [0,1]$ sampled in step 5 tunes estimation from ordinary least squares ($\psi_{k}=0$) to weighted least squares ($\psi_{k}=1$) to allow for higher noise levels associated with higher expression levels.

#### 2.2.2 Prior distributions

A natural conjugate choice of prior distribution for the error variance $\sigma_{k}^{2}$ is the scaled inverse-$\chi^{2}$ distribution with $n_{k,0}$ degrees of freedom and scale $\sigma_{k,0}^{2}$, while a continuous uniform prior, U([0,1]), can be used for the parameter *ψ_k_*. The latter results in a full conditional posterior distributions that can be numerically computed. Alternatively, a full conditional least squares estimate of *ψ_k_* can be derived (see SI section 4). A gamma prior distribution is used for the degradation rate *δ*, while the number of regulators, *ν*, is assumed to be Poisson with parameter *λ*.

Prior distributions are also formulated for the set of transcription factors, Φ, and their threshold levels, $\rho_{\phi }$. Here, we consider two scenarios motivated by the real data examples discussed below, but we emphasize that in principle any other proper distribution formulated based on external knowledge can be used. In the first scenario we assume that there is very little prior information and use uniform prior distributions for the set of regulators and for the threshold levels. The second scenario assumes that transcriptional switches are more likely to be caused by substantial changes in the TF levels. Hence, the overall dynamics of any candidate regulator, as quantified by the range of its (smoothed) profile across experiments, are used to compute the prior for the set of TFs and the temporal dynamics, as quantified by the gradient of the smoothed profile, are employed for the prior of the threshold levels (see SI section 3 for details).

## 3 Simulation studies

### 3.1 Repressed activation network

In this simulation study, a target gene is assumed to be observed simultaneously with six candidate TFs in two experimental conditions over a period of *L* = 10 hours with measurements recorded every about 0.5–1.5 hours and 4 replicates per experiment to reflect a realistic sample size scenario. The simulated profiles of the TFs and the target gene are the sum of a deterministic profile, Ωh(t) ($\Omega =10^{3}$), and normal measurement error with standard deviation $\sigma =20\sqrt{\Omega }$ imposing slightly higher noise levels compared to the real data considered in the next section.

The regulation of the target gene is the repressed activation shown in Figure 1. The profiles, *h*(*t*), of the first two candidate TFs, *f*_1_ and *f*_2_, are the same as in Figure 1, but four alternative TFs and the target gene itself are also considered here as candidate TFs. The third candidate *f*_3_ has the same profile as *f*_2_ in experiment 2, but unlike *f*_2_, its profile is also the same in experiment 1. The fourth TF *f*_4_ has constant low levels in the first experiment and constant high levels in the second experiment and could potentially explain differences between all transcription rates of the target gene of each experiment. Candidate *f*_5_ is a reflection of *f*_2_ and thus it provides an alternative regulation model in which *f*_1_ and *f*_5_ are AND activators (i.e. both are present for activation) of the target. Finally, the profile of *f*_6_ has a similar form to *f*_1_ but with a smaller range.

The profiles, *h*(*t*), described above are derived as solutions of an ODE system with transcription of the target being regulated by *f*_1_ and *f*_2_ through Hill Functions of the nuclear concentration of the TF protein levels (for more details see SI section 5.1). However, we need to adapt to the case that only the mRNA expression levels of the candidate TFs and the target are observed as is the case in many current experimental protocols including microarrays. In order to apply the TRS methodology to these datasets, it is necessary to approximate the protein profiles of the candidate TFs from their mRNA expression levels. We do this by fitting splines to derive a continuous mRNA expression profile and using Wild bootstrap (Wu, 1986) to characterize the noise levels around the smoothed expression. Then, an ODE model could be used to derive the protein profiles from the smoothed expression profiles, but this requires knowledge of the translation and protein degradation rate. Another approach is based on the standard assumption in reverse engineering that the TF protein level is a delayed or linear function of its mRNA expression level. Supplementary Figure S1–S2 in SI provide examples of the derivation of the expression profile $P_{f}(t)$ using both of these methods. This issue is discussed further in SI section 2.

Informative prior distributions are constructed as described above (see SI Supplementary Fig. S6). The RJMCMC algorithm is run for 1M iterations with execution time about 1.25 hours (2.5-GHz Intel Core i7 processor). The algorithm appears to quickly converge (see SI Supplementary Fig. S8). The result of the posterior inference using our RJMCMC algorithm are summarized below.

The algorithm assigns the largest posterior probability 0.79 to models with two TFs and a probability of 0.18 to models with three TFs. The estimate of the posterior probability of TF *f*_1_ to be in Φ is approximately 0.72 with its alternative TF *f*_6_ given a smaller probability 0.30 as it is a priori less preferable (if no prior information is imposed on the TF set, the probabilities are 0.44 and 0.37 see SI Supplementary Fig. S11). The inhibitor *f*_2_ has posterior probability near 1, with its alternative *f*_5_ being much less sampled as it has a more noisy profile. The other candidates *f*_3_ and *f*_4_ as well as the target (autoregulation) have much smaller posterior probabilities. The set $\left\{f_{1},f_{2}\right\}$ has the biggest posterior probability (0.56), while $\left\{f_{2},f_{6}\right\}$ has probability 0.23 and other regulation sets much smaller probabilities. The fit of the TRS model to the simulated data under these regulation sets is excellent (see SI Supplementary Fig. S9).

Results for the case of non-informative priors applied to the same choice of TF and their threshold and for a Poisson prior on the number of TFs with larger mean parameter (*λ* = 1) are given in the SI. They demonstrate the importance of using a small parameter value for the Poisson prior to avoid overfitting and the use of informative priors on the TF set and their thresholds for attaining more robust results.

We also apply the GRNInfer tool (Wang *et al.*, 2006) to the same simulated data. The tool runs extremely fast, with computational time a few seconds, and provides estimates for the regression coefficients for each of the candidate TFs which constitutes more limited information than this provided in TRS. In this study, the GRNInfer tool detects the activation of the target but picks *f*_6_, the alternative of the true regulator *f*_1_, as the activator (coefficient 0.85). A large coefficient (–1.3) is allocated to the target gene, but the tool cannot conclude whether this is due to degradation or self-regulation. The tool also fails to detect the repression of this activation caused by *f*_2_ with all coefficients except for the target and *f*_6_ in (−0.05,+0.05) (see also SI Sect. 5.1.7).

### 3.2 SOC1 transcription regulation

**Fig. 3.**
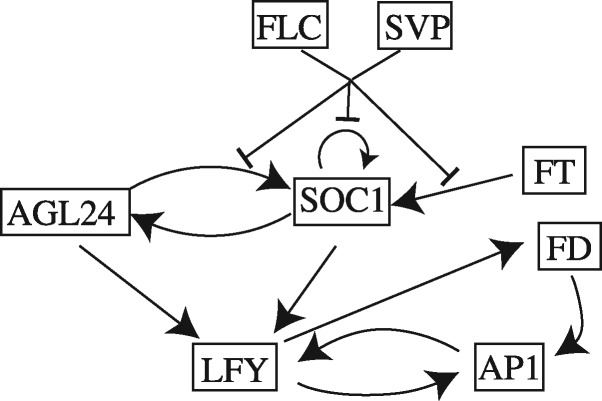
Diagram of the flowering time of *A. thaliana* system (Leal Valentim *et al.*, 2015)

We also consider the complex regulation of the SUPPRESSOR OF OVEREXPRESSION OF CONSTANS (SOC1) gene in the system related to flowering time published in Leal Valentim *et al.* (2015). The gene SOC1 is regulated by five TFs, while three more genes are part of the network (see Fig. 3).

In a similar fashion to the example in the previous section, we simulate data in four experimental conditions where the system is assumed to be observed for 10 days with 0.5–1.5 days observation frequency. The simulated profiles are again the sum of Ωh(t) ($\Omega =10^{3}$), where *h*(*t*) corresponds to solutions of an ODE system (see SI sect. 5.2) and normal measurement error with $\sigma =20\sqrt{\Omega }$. The simulated mRNA expression levels of all genes are used to approximate the TF profiles using splines and the Wild bootstrap method as above. Informative prior distributions are constructed and the TRS RJMCMC is run for 1M iterations in about 2.5 h. The increased execution time compared to the first simulation study is due to the larger amount of data and the larger regulation sets.

The algorithm detects the true TFs with very high probabilities with some of the other genes also being sampled. The most likely model is the true regulator set with the regulator set that does not include SOC1 also receiving higher probability (0.43 and 0.20, respectively) than other regulator sets. The true interactions can be correctly deduced from the TRS algorithm and the data fit is again excellent (see SI Supplementary Fig. S21).

We also apply the GRNInfer tool to the same data. The tool incorrectly allocates the largest coefficient (1.59) to the FD gene that is not a TF. The repressor SVP and AP1, which is not a TF, receive a relatively large negative coefficient (–0.42 and –0.43, respectively). The other genes receive smaller coefficients with exception the target gene SOC1 (–0.80), but the tool is unable to infer whether this is due to auto-suppression or degradation (see also SI Sect. 5.2.4).

The two simulation studies clearly demonstrate that the TRS algorithm is well able to detect the correct regulation model, along with possible alternative regulation models, under realistic noise levels and sample sizes compatible with our observed data for *A.thaliana*.

## 4 Application to *Arabidopsis thaliana*

Microarray technology was used to extract mRNA expression profiles of the response of *A.thaliana* to multiple biotic stresses, namely *Botrytis cinerea* (Windram *et al.*, 2012), *P.syringae* hrpA and *P.syringae* DC3000 (Lewis *et al.*, 2015). The response to *Botrytis* was observed in 4 replicates every 2 hours over a period of 48 hours, while for *P.syringae* hrpA and DC3000 the period was 13.5 hours, with observation frequencies ranging from 1 to 2.5 hours.

We focus on two target genes of interest, namely Arabidopsis NAC 092 (ANAC092, ORE1) (Du *et al.*, 2014) and SCARECROW-LIKE 3 (SCL3, Heo *et al.*, 2011), which were differentially expressed in the observed stresses. A number of potential TFs were identified through Y1H technology. Specifically, twenty candidate regulators were identified for ANAC092, among them three from the TCP family, two from the ERF family and three from the Arabidopsis NAC family. For SCL3, fifteen regulating genes were identified, which include three from the TCP family and two from the ERF family. All gene names and GST IDs are derived from CATMA database (Crowe *et al.*, 2003) and provided in SI Sect. 6.1.

To approximate the protein profiles of the candidate TFs from their mRNA expression observed in the microarray experiments, we fitted splines and used the wild bootrstap method as above (see SI sect. 2). We constructed informative prior distributions, as described in previous sections (see SI Supplementary Figs S22–S26), for the set of transcription factors and their threshold levels. In both cases a Poisson prior (λ=0.15) is used for the number of transcription factors, a vaguely informative scaled $\chi^{2}$ distribution ($n_{k,0}=0.001,\;\sigma_{k,0}^{2}=0.001$) is used as a prior for the precision $\sigma_{k}^{-2}$ and an informative Gamma distribution with mean 0.345 corresponding to an approximate half life of 2*h* is used to specify the degradation rate *δ* (sd 0.1543). The trans-dimensional MCMC algorithm described in earlier sections is run for 1M iterations (execution times 2.6 and 3.2 hours).

**Fig. 4.**
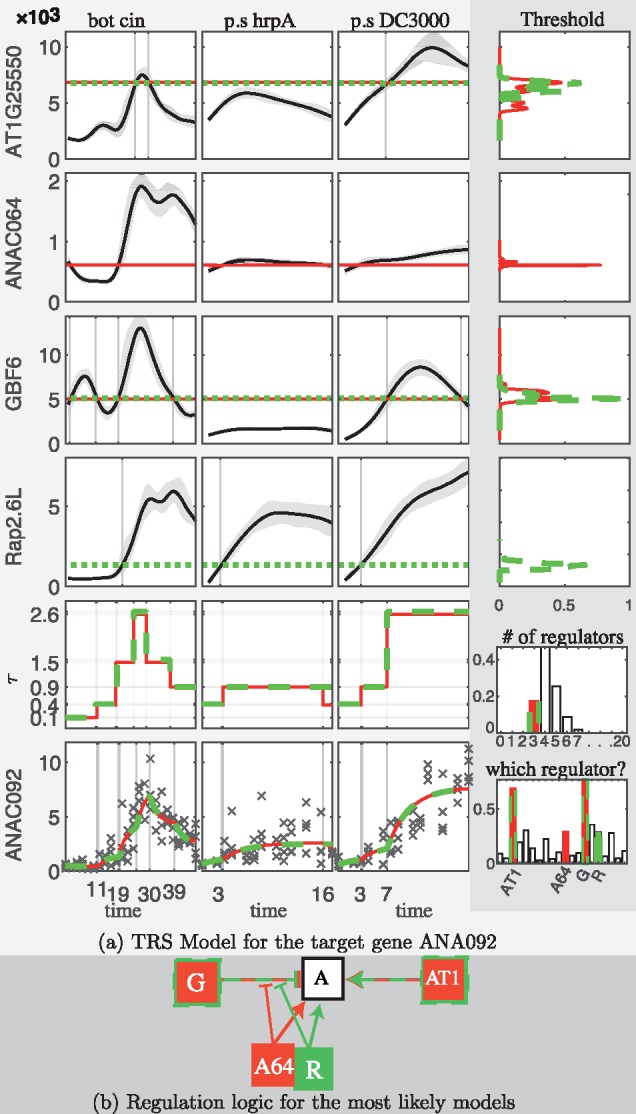
Posterior inference for the TRS model of the target gene ANAC092. (**a**) The smooth protein profiles (solid line) along with the estimated threshold (dotted line) of the TFs AT1G25550 (AT1), ANAC064 (A64), GBF6 (G), Rap2.6L (R) under two of the *a posteriori* most likely models (red and green colors) are displayed in the first four rows of the three panels on the left (*Botrytis*, *P. syringae* hrpA and DC3000, respectively). The right panel shows the estimated posterior density (first 4 rows) for the threshold level of each TF with units linking to the level of threshold in the other panels and the posterior probabilities for the number of TFs (fifth row) and for the candidate to be involved in any regulation model (last row). The three panels on the left of the fifth row give the estimated transcription profiles of the target gene and in the bottom row the observed (crosses) and fitted (red solid and green dashed line) mRNA profile of the child gene under the two most likely models. (**b**) The regulation diagram summarizes the logics of these TRS models (Color version of this figure is available at *Bioinformatics* online.)

The following results were obtained for posterior inference. For gene ANAC092 (see Fig. 4) the posterior probability of having 4 regulators, π(ν=4|y), out of 20 candidates was estimated to be 0.47, while π(ν=3|y)≈0.17 and π(ν=5|y)≈0.25. The candidates GBF6 and AT1G25550 received high posterior probabilities to be regulators, while TCP21, ANAC025, ANAC064, Rap2.6L and AT5G58900 also received higher posterior probabilities than the rest of the candidates (0.77,0.70,0.36,0.30,0.30,0.29,0.28, respectively). The pair of candidates GBF6 and AT1G25550 have much larger posterior probability (0.52) to be part of the regulators compared to other pairs (≤0.25), with AT1G25550 activating the target both in *Botrytis* and *P.syringae* DC3000 and GBF6 acting as a repressor. The candidates TCP21, ANAC025, ANAC064, Rap2.6L are sampled alternatively to block the repression caused by GBF6. Such hypothesized interactions could be tested by biologists in additional experiments.

**Fig. 5.**
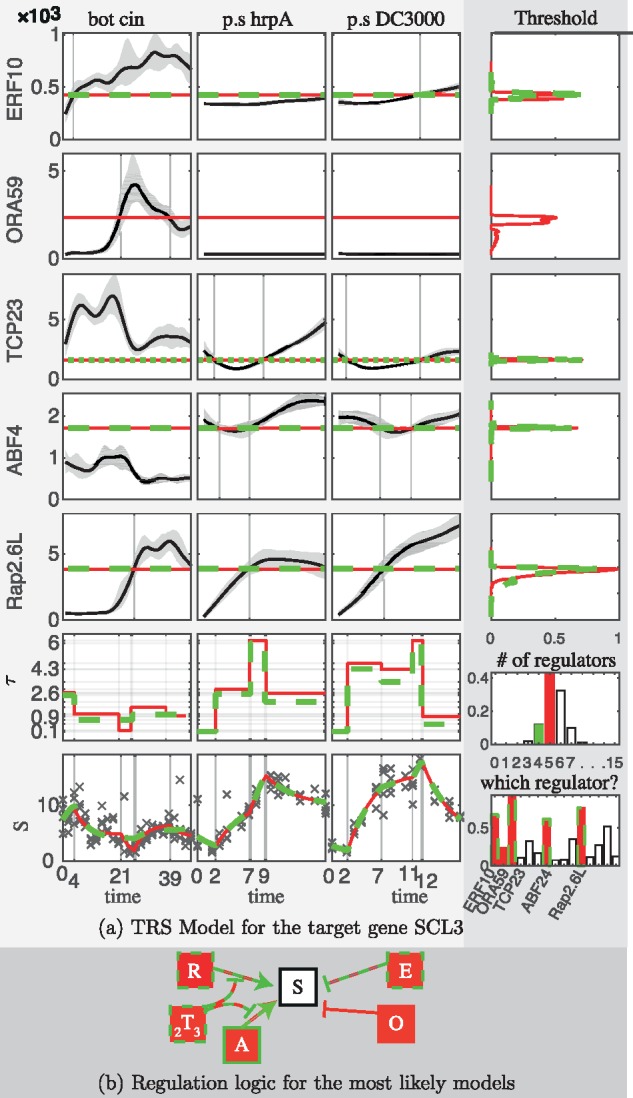
Posterior inference of the TRS model of the target gene SCL3. The setup of the panels is the same as in Figure 4 showing the 5 regulators, ERF10 (E), ORA59 (O), TCP23 (_2_T_3_), ABF4 (A) and Rap2.6L (R) of the two most likely models (red and green colors) (Color version of this figure is available at *Bioinformatics* online.)

Regarding the SCL3 gene (see Fig. 5), the posterior probability of having 5 regulators, π(ν=5|y), out of 15 candidates was estimated to be 0.43, while π(ν=6|y)≈0.32. High posterior probabilities were estimated for the candidates TCP23, Rap2.6L, ERF10 and ABF4 (0.93,0.77,0.68,0.62, respectively) to be regulators. The couple of regulators TCP23 and Rap2.6L are part of the regulator set with probability 0.74, while the triplet that also includes ERF10 with probability 0.42. The most likely regulation logics suggest that ERF10 is a repressor, while Rap2.6L and ABF4 are activators with their activation being repressed by TCP23. ORA59 is also a repressor on the second most likely regulation logic.

## 5 Discussion

In this study we suggest the use of the TRS model as a simple biologically interpretable model to draw inference about possible regulatory logics between a set of putative TFs and a gene of interest. Assuming that any interactions are consistent across different experiments and over time imposes constraints that, in principle, allows us to identify the set of regulatory TFs and to deduce their dynamic regulation logic. The algorithm for Bayesian inference on the TRS model parameters is trans-dimensional and is efficiently solved by the suggested RJMCMC. The advantage of this methodology is that different combinations of parents are sampled within the algorithm allowing us to identify a set of all plausible regulation models that are compatible with the data and to rank them according to their posterior model probabilities.

We showed that the methodology works well for two simulation studies with realistic sample sizes and noise levels, and present results of its application to the transcriptional regulation of two target genes of Arabidopsis Thaliana under multiple biotic stresses. The algorithm indeed suggests a few alternative regulation models and it is clear that despite its simplicity it can infer regulation logics to a greater degree of complexity than existing methods.

We note that further checks against a potentially much larger set of parents can be carried out to see if other potential TFs exist, which may not have been included in the set of candidate parents and which may have similar transcription profiles across experiments. Such genes can be identified by performing a cluster analysis across experiments as, for example, suggested in Polanski *et al.* (2014). In the case of our *A. thaliana* data examples the clusters of interest only contained very few TFs with jointly similar profiles and most of them could be ruled out on the basis of other biological information. However, if combined with a cluster analysis of the TFs across experiments as a pre-processing step, the TRS methodology has the potential to be applied to very large sets of putative TFs.

In order for the methodology to work well it is essential that the magnitude of the observations between experiments has comparable scales. In cases where the measurements are not comparable, an additional computation is necessary to bring them to some relative measurement unit.

Finally we note that an extension to a network methodology with multiple targets considered simultaneously is within reach and can be built on the suggested TRS approach.

## Supplementary Material

Supplementary Data
