# Supplementary material for: Inferring transcriptional logic from multiple dynamic experiments
Source: Bioinformatics. 2017 Jun 28;33(21):3437–44. doi: 10.1093/bioinformatics/btx407 (PMC5860162; doi:10.1093/bioinformatics/btx407)
Supplement: Supplementary Data [file btx407_si.pdf]

Supplementary Information for the article: “Inferring transcriptional logic from multiple dynamic experiments”

Giorgos Minas, Dafyd J Jenkins, David A Rand and Bärbel Finkenstädt

Abstract

In this note we provide further details and discussion about: (i) alternative regulation models, (ii) the approximation of the TF protein profile, (iii) prior distributions, (iv) the parametric weighted least squares method (v) the two simulation studies (vi) the application to *A. thaliana* data. We refer to the main paper ”Inferring transcriptional logic from multiple dynamic experiments” by **I** and this supplementary information by S.

Contents

1 Alternative regulation models **2**

2 Approximation of TF protein profile from mRNA expression data **4**

3 Prior distributions **8**

3.1 Set of regulators 9

3.2 Threshold level of regulators 10

4 The parametric weighted least squares method **11**

5 Simulation studies **13**

5.1 Repressed activation network 14

5.1.1 Simulated data 18

5.1.2 Prior distributions 18

5.1.3 Monte Carlo Markov Chains of various parameters 21
5.1.4 Inference 22
5.1.5 Results for less informative prior distributions 23
5.1.6 Results for smaller values of smoothing parameter 27
5.1.7 Output of the GRNInfer tool 31
5.2 Flowering time network 31
5.2.1 Simulated Data 36
5.2.2 Prior distributions 36
5.2.3 Monte Carlo Markov Chains of various parameters 38
5.2.4 Output of the GRNInfer tool 39

6 Application to *A. Thaliana* **40**
6.1 Target and regulator gene names 40
6.2 Prior distributions 42
6.3 Monte Carlo Markov Chains of various parameters 48

1 Alternative regulation models

In this section we discuss various alternative regulation models that are used to describe the transcription rates of target genes in the current literature. We first consider the models that describe the transcription rate, τ, of a target gene as a regression function of the regulators' profiles. The general form of those models is

$$\tau(t) = f(\boldsymbol{\beta}; p_1(t), p_2(t), \ldots, p_n(t)), \quad t \in [0, L],$$

where $\boldsymbol{\beta}$ a parameter vector and $p_1(t), p_2(t), \ldots, p_n(t)$ the regulators profiles. Two commonly used models of this type (see Madar et al. [2010]; Yip et al. [2010]; Huynh-Thu et al. [2010]; Wang et al. [2006]; Ou-Yang et al. [2017]) are the linear model,

$$\tau(t) = \beta_0 + \sum_{i=1}^{n} \beta_i p_i(t)$$

and the non-linear model

$$\tau(t) = \frac{\beta_0}{1 + \exp(-\beta_0 - \sum_{i=1}^{n} \beta_i p_i(t))}.$$

Note that in both models the effects of the two regulators in the transcription rate are additive. That is, the terms corresponding to each regulator are added to derive a weighted sum of their individual effects with weights controlling their effect to the value of the transcription rate.

Consider the example in Fig. **I**1 where two regulators form a repressed activation regulation network and suppose for simplicity that the regulator profiles are reduced to binary. Then the four states of the regulation model are given in Table 1.

p_1	p_2	τ
0	0	τ_0
0	1	τ_0
1	0	$\tau_0 + \tau_1$
1	1	τ_0

Table 1: Logic table of the repressed activation regulation model. Here p_1 is the profile of the activator and p_2 the profile of the repressor of this activation.

Here the transcription rates are such that τ_1 is much larger than the basal rate $\tau_0 > 0$. We can easily see that the linear model is not able to describe the above regulation. For instance, if β_2 is taken to be $-\beta_1$, then the model would fit for all states except $p_0 = 0, p_1 = 1$.

Note that the same principle applies to any regulation mechanism where interactions between the regulators are involved. For example AND activations as in Table 2 cannot be described by the linear model. It is easy to extend the case for continuous scale profiles.

p_1	p_2	τ
0	0	τ_0
0	1	τ_0
1	0	τ_0
1	1	$\tau_0 + \tau_1$

Table 2: Logic table of the AND activation regulation model.

Similarly, AND repressions, XOR activations or repressions and other regulation mechanisms that involve interaction between multiple regulators cannot be described by the above linear model. It is important to stress here that the above limitation does not arise because

of the linear relation of τ with p_1 and p_2, but because the terms that describe the effect of each regulator to τ are added to obtain the transcription regulation model. The non-linear model described above cannot model the above interactive regulation mechanism. It can only model non-interactive non-linear regulations. For example, the non-linear model can describe regulation of a target gene by two regulators that independently activate the target (say from a different binding site) and they also appear to cooperate and produce some additional activation of the target when both are present.

It is necessary to add an interaction term to the model in order to describe interactive regulation using this type of regression model. That is, for the linear model with two regulators, we can use the following model of the transcription rate function

$$\tau(t) = \beta_0 + \beta_1 p_1(t) + \beta_2 p_2(t) + \beta_{12} p_1(t) p_2(t).$$

However, the inclusion of the interaction term increases the number of parameters of the model and this can be prohibiting when the model involves a large number of regulators. In particular, the four parameters of the latter model would not be identifiable unless sufficient amount of data is observed from all four combinations of different activation states.

Another approach is to use a Boolean logic model that can describe interactive regulations. However, the Boolean logic would not be able to distinguish between regulators with different strength of the interaction. The arbitrariness of the discretisation algorithm which is performed as a pre-processing step in methods of statistical inference based on Boolean logic models (see for example [Bornholdt, 2008; Han et al., 2014]) can also be problematic, particularly in cases where the switches of the target gene from 0 (OFF) to 1 (ON) states and vice versa are not clear in the continuous scale.

2 Approximation of TF protein profile from mRNA expression data

In most experimental protocols that are currently used for inferring transcription regulation, the putative regulators are observed simultaneously with target genes only in terms of their

mRNA expression level. The TF protein level is typically not observed simultaneously with the target mRNA expression. It is generally accepted that in eukaryotic organisms mRNA of regulating genes first translate to protein before regulating the target's transcription. As the TF protein level is typically unobserved, it has to be approximated using mRNA expression. One standard model for protein translation is the following simple ODE

$$\dot{P}(t) = \alpha M(t) - \delta_P P(t) \tag{1}$$

that describes the translation of the mRNA expression $M(t)$ at rate α to protein $P(t)$ that also degrades with rate δ_P.

More complicated models are also commonly used. In particular, a commonly used model for eukaryotic transcription takes into account that the translated protein is first located in the cytoplasm and then moves to the nucleus to initiate transcription. Export of the protein from the nucleus to the cytoplasm is also possible while the protein can degrade both in the cytoplasm and the nucleus. These reactions can be described by the following ODEs

$$\begin{aligned} \dot{Pc}(t) &= \alpha M(t) - \delta_P Pc(t) + k_e Pn(t) - k_i Pc(t), \\ \dot{Pn}(t) &= k_i Pc(t) - k_e Pn(t) - \delta_P Pn(t), \end{aligned} \tag{2}$$

where $Pc(t)$ and $Pn(t)$ the cytoplasmic and nuclear concentrations, respectively, and k_i and k_e the import and export rates, respectively. This model is used for producing the simulated data in our examples (see Sect. 5).

On the other hand, a simple approach is to assume that the TF profile is simply a delayed version of the mRNA expression profile and take

$$P(t) = M(t + h) \tag{3}$$

where h the delay time. This is an implicit assumption used in many reverse-engineering methods (e.g. Madar et al. [2010]; Yip et al. [2010]; Huynh-Thu et al. [2010]; Wang et al. [2006]; Ou-Yang et al. [2017]) that use the mRNA expression level of the regulating gene to

describe the regulation of the target gene transcription rate.

In the examples used below, we consider both the ODE model in (1) and the delay model (3) with fixed parameter values for approximating the TF protein profiles $P_f(t)$ of the candidate regulators.

To derive the mRNA expression profile, $M(t)$, of the regulating genes from the observed data, we use a smoothing spline kernel. More specifically, we use the MATLAB 2016b function

```
f = fit(timepoints,data,'smoothingspline', 'SmoothingParam', s);
```

where s the smoothing parameter, $s \in [0, 1]$.

To characterise the noise levels of the mRNA expression, we use the Wild bootstrap method Wu [1986]. In summary, a smoothing spline, $s(t)$, is fitted to observations $Y(t_i)$, $i = 1, 2, \ldots, n$. The residuals $e_i = Y(t_i) - s(t_i)$ are obtained and used to repeatedly generate normally distributed errors, $\hat{e}_i = e_i Z$, where $Z \sim N(0, 1)$. These are used to compute bootstrap samples $\hat{y}_i^{(b)} = \max(0, s(t_i) + \hat{e}_i)$, $i = 1, 2, \ldots, n$, $b = 1, 2, \ldots, B$. Smoothing splines $\hat{s}(t)$ are fitted to each of these bootstrap samples which gives a sample of smoothing splines. As we explain below, this bootstrap samples are used for constructing informative priors. As we discuss in **I**, the use of these priors is optional.

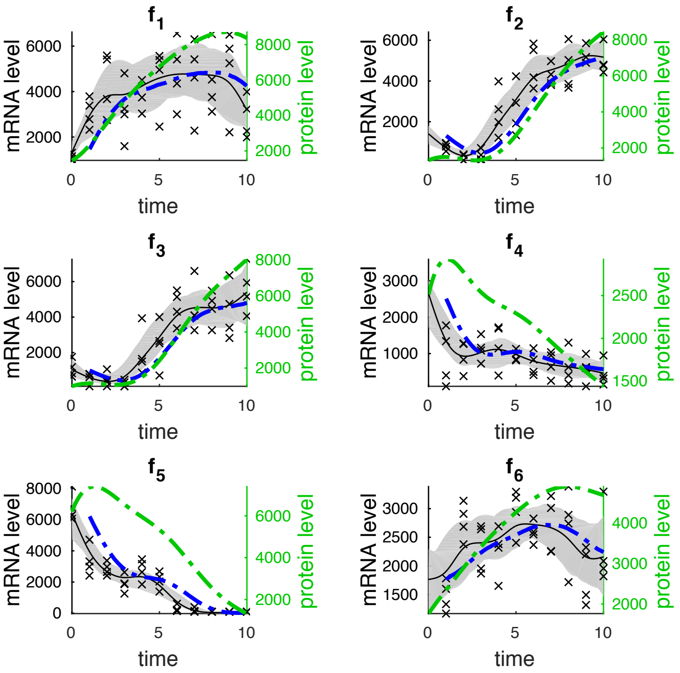

Figure 1: **Computation of the regulator expression P_f from mRNA expression profiles.** In this example, four samples (crosses) of the mRNA expression levels of the candidate regulators, $f_1, f_2, \ldots, f_6$, are observed in each time point. The observed mRNA expression profiles are smoothed using a spline kernel (black solid line) and Wild bootstrap was used to derive samples of the smoothing spline (grey area corresponds to 90% confidence envelopes). The smoothing spline is either delayed (blue dashed line) by h hours ($h = 1$) or imposed to the protein model in (1) to predict the protein levels (green dashed line). For the latter case, the value of the translation rate here is equal to $ln(2)/1$ and the protein degradation rate $ln(2)/2$ (half-life 2 hours).

In the above figure, the smoothing parameter s is set equal to 0.5. To examine the stability of the approximation in varying values of $s \in [0, 1]$ we also consider the more extreme values of s, $s = 0.1$ and $s = 0.9$. The results are displayed in Fig. 2. Despite the small local changes in the profiles, the overall form of the profile is preserved for all three values of s considered here.

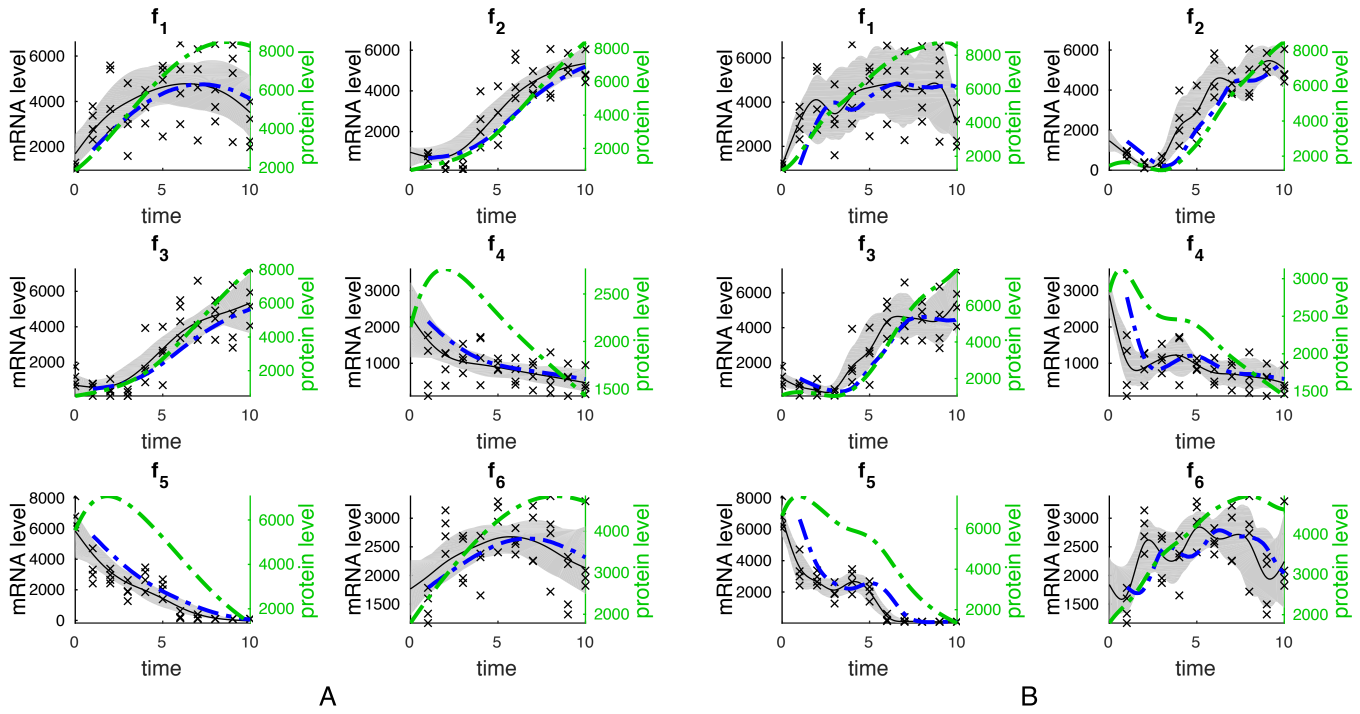

Figure 2: **Computation of the regulator expression P_f from mRNA expression profiles for different values of the smoothing parameter $s = 0.1$ (panel A) and $s = 0.9$ (panel B)**. The setup of the figure is the same as in Fig. 1.

We run the simulation studies and the real data examples with $s = 0.1$ because we wish to eliminate noisy local changes in the profiles of the candidate regulators. However, in Section S5, we provide the results of running the TRS algorithm with $s = 0.5$.

3 Prior distributions

We begin by describing the informative prior distributions for the set of regulators and their threshold levels briefly introduced in **I**.

As discussed in Sect. **I**2.2, these prior distributions are derived under the assumption that during an experiment candidates with more dynamic profiles are more likely to be regulators than candidates with less dynamic or "flat" profiles. Similarly, transcriptional switches are more likely to be caused by substantial changes in the regulator levels than by small noisy changes. The overall dynamics of any candidate regulator are measured by the range of its smoothed expression profile across experiments while the gradient (first derivative) is used to measure the temporal dynamics at any given time. We account for nonlinear effects such

as saturation (i.e. effect of larger values saturates after some high level) by applying sigmoid functions, $S_1(\cdot)$ and $S_2(\cdot)$ to the range and gradient value, respectively. The noise levels of the observed regulators profile are taken into account by standardizing the value of the range and gradients with respect to their variance estimated by the Wild bootstrap method [Wu, 1986].

The prior probability, $\pi(f \in \Phi)$, of each of the candidate regulators, $f \in \mathcal{F}$, to be a regulator is set proportional to the sigmoid function, $S_1(\cdot)$, of the length of its standardized range (see section S3.1 for more details). To derive the prior distribution $\pi(\rho_\phi)$ of any given value of the threshold level, ρ_ϕ, in the profile range, R_ϕ, of the regulator ϕ, we first derive the intersections of the constant line ρ_ϕ to the profile, $P_\phi(t)$, across the multiple experiments. If a single intersection is derived, we compute the value of the gradient at this intersection and standardize it based on the bootstrap estimate of variance. If there are multiple intersections, the minimum of their standardized gradient values are derived to penalise threshold levels which give any crossings at non-dynamic levels. The prior distribution $\pi(\rho_\phi)$ is then set proportional to the sigmoid function $S_2(\cdot)$ of the minimum value of the standardized gradients at the intersections produced by ρ_ϕ unless a range with no intersections exists. Such ranges occur if multiple experiments are considered and the regulator consistently lies higher in one experiment and lower in another. Such differences may explain changes in transcriptional activity of the target gene, particularly, if the length of these "gap" ranges is substantial. In case such ranges occur in R_ϕ of regulator ϕ, the prior distribution $\pi(\rho_\phi)$ is a continuous mixture distribution with uniform mixture component for the gap ranges and the above gradient-based distribution for the covered ranges. The mixture weight for each range is set proportional to the length of its range (see section **S**3.2 for more details).

3.1 Set of regulators

Let $P_f^{(k)}(t)$, $t \in [0, T]$ be the (smoothed) profile of a candidate regulator $f \in \mathcal{F}$ in experiment k, $k = 1, 2, \ldots, K$. We consider the length of the range of the regulator profile across experiments $L_f = \max_{t,k} P_\phi^{(k)}(t) - \min_{t,k} P_f^{(k)}(t)$. We use the Wild bootstrap method [Wu, 1986] to derive

an estimate of its standard deviation σ_{L_f}. The standardised range is then

$$\bar{L}_f = \frac{L_f - L}{\sigma_{L_f}}$$

where L is the median range of all candidate regulators. A sigmoid function $S_1(\cdot)$ is applied to $\bar{L}_f$. Here we use the cumulative distribution function of the Gaussian distribution with mean and variance respectively equal to the overall median and variance of $\bar{L}_f$ for all candidates $f \in \mathcal{F}$. The variance is multiplied by a non-zero factor (possibly equal to one) to allow for increasing or decreasing non-linearities and saturation effects. The prior probability for each candidate, $f \in \mathcal{F}$, to belong to the regulator set Φ is then

$$\pi(f \in \Phi) \propto S_1(\bar{L}_f), \quad \sum_{f \in \mathcal{F}} \pi(f \in \Phi) = 1.$$

These probabilities are used to derive the prior probability for each regulator set $\Phi = \{\phi_1, \ldots, \phi_\nu\}$

$$\pi(\Phi) = \sum_{(\phi_1, \phi_2, \ldots, \phi_\nu) \in \mathcal{P}(\Phi)} \pi_0(\phi_1) \frac{\pi_0(\phi_2)}{1 - \pi_0(\phi_1)} \cdots \frac{\pi_0(\phi_\nu)}{1 - \pi_0(\phi_1) - \cdots - \pi_0(\phi_{\nu-1})}$$

where $\mathcal{P}(\Phi)$ the set of permutations of the elements of the regulators set Φ.

3.2 Threshold level of regulators

Let $g_f^{(k)}(t)$ be the gradient (first derivative) of the expression, $P_f^{(k)}(t)$, of the candidate regulator $f \in \mathcal{F}$ at time $t \in [0, L^{(k)}]$ of experiment k. Using the Wild bootstrap method, we derive a sample of $g_f^{(k)}(t)$ for each time-point, t, in a dense partition $(0, t_1, t_2, \ldots, t_{p-1}, L^{(k)})$ of the observed time interval $[0, L^{(k)}]$ of experiment k, $k = 1, 2, \ldots, K$. Using this sample we derive an estimate, $\sigma_{g_f^{(k)}}(t)$, of its standard deviation. This gives the standardized gradient (accounting for noise in the observed profile)

$$\bar{g}_f^{(k)}(t) = \frac{g_f^{(k)}(t) - g}{\sigma_{g_f^{(k)}}(t)}, \quad t \in [0, L^{(k)}]$$

where g the overall median gradient value across $\mathcal{F}$ and over time.

Take a threshold level ρ_f and let $\mathcal{C}_{\rho_f}$ be the set of crossings of the constant line at ρ_f on the profiles $P_f^{(k)}(t)$, $k = 1, 2, \dots, K$. This set is finite with probability 1 for noisy profiles $P_f^{(k)}(t)$. If it is non-empty, take $p(\rho_f) = S_2(\min_{c \in \mathcal{C}_{\rho_\phi}} \bar{g}_f(c))$, where $S_2(\cdot)$ the Gaussian cumulative distribution function with mean g and standard deviation the overall median of $\sigma_{g_f^{(k)}}(t)$ across $f \in \mathcal{F}$, $t \in [0, L^{(k)}]$, $k = 1, 2, \dots, K$. We also set a cut-off value, $\bar{g}_{min}$, for the standardised gradient, so that if $\min_{c \in \mathcal{C}_{\rho_\phi}} \bar{g}_f(c) < \bar{g}_{min}$, then $p(\rho_f) = 0$. This allows for eliminating the possibility of setting thresholds at levels which the changes are too small for causing switches in the target's transcription. If no between-experiment gaps are observed in the profile range, R_f, of f, that is $\mathcal{C}_{\rho_f} \neq \emptyset$, for all $\rho_\phi \in R_\phi$, the prior probability distribution $\pi(\rho_\phi)$ is

$$\pi(\rho_f) \propto p(\rho_f), \qquad \int_{r \in R_f} \pi(r) dr = 1$$

If gap ranges exist, that is $\mathcal{C}_{\rho_f} = \emptyset$, for at least one $\rho_f \in R_f$, compute the length of the ranges, $l_f^{(1)}, \dots, l_f^{(m)}$, of each of the gap and covered ranges, $R_f^{(1)}, \dots, R_f^{(m)}$. The prior probability distribution $\pi(\rho_f)$ is then a continuous mixture with weights equal to $l_f^{(i)} / \sum_{j=1}^m l_f^{(j)}$ for $\rho_f \in R_f^{(i)}$ and mixing components either a uniform distribution in $R_\phi^{(i)}$ if the latter is a gap range or the gradient-based distribution computed above, but restricted in $R_\phi^{(i)}$, $i = 1, \dots, m$.

4 The parametric weighted least squares method

To express heterogeneity across observations, we set the observation variance of experiment k, $k = 1, 2, \dots, K$, as $\sigma_k(t) = \sigma_k v_k(t)$, where $v_k(t) = (w_k(t))^{-\psi_k}$, with $w_k(t)$ some fixed time-dependent function and $\psi_k \in [0, 1]$ an unknown parameter. In matrix form, the vector of time-series observations $\boldsymbol{y} = (y_k(t_i))$ has covariance Σ which is a block-diagonal matrix with blocks

$$\Sigma_k = \sigma_k^2 Q_k$$

and Q_k a diagonal matrix with main diagonal entries equal to $v_k(t_i)$, $i = 1, 2, \dots, n_k$. Let Q be the block diagonal matrix with blocks Q_k.

Define the vector of parameters $\boldsymbol{\beta} = (M_1(t_0), \dots, M_K(t_0), \tau_{\alpha_1}, \dots, \tau_{\alpha_q})$ with $M_k(t_0)$ the initial values of experiment k and τ_{α_j} the transcription rates associated with regulator activation

state α_j, $j = 1, \ldots, q$. Let X be the matrix of covariates of the linear model in (**I**2). Assume first that ψ_k, $k = 1, \ldots, K$, is fixed and thus Q is also a fixed matrix. Then, weighted least squares (wls) is performed as ordinary least squares (ols) but X and $\boldsymbol{y}$ are replaced by $Q^{-1/2}X$ and $Q^{-1/2}\boldsymbol{y}$, that is

$$\hat{\boldsymbol{\beta}} = V_{\boldsymbol{\beta}} X Q^{-1} y, \qquad V_{\boldsymbol{\beta}} = (X^t Q^{-1} X)^{-1}$$

and

$$s_k^2 = \frac{1}{n_k - q_k - 1} (\boldsymbol{y}_k - \hat{\boldsymbol{M}}_k) Q_k^{-1} (\boldsymbol{y} - \hat{M}_k).$$

where $\boldsymbol{y}_k = (y_k(t_1), \ldots, y_k(t_{n_k}))$ and $\hat{\boldsymbol{M}}_k = (M_k(t_1), \ldots, M_k(t_{n_k}))$ the observed and predicted (with parameter values $\hat{\boldsymbol{\beta}}$) mRNA profile of the target gene in experiment k and q_k the number of distinct transcription rates in experiment k, $q_k \leq q$. After computing the above, we can derive the full conditional posterior distributions $\sigma_k^2 | \boldsymbol{\beta}, \boldsymbol{y}$ which are scaled inverse-χ^2 distributions with $n_{k,0} + n_k$ degrees of freedom and scale parameters $(n_{k,0}\sigma_{k,0}^2 + (n_k - q_k - 1)s_k^2)/(n_{k,0} + n_k)$. This is used in step 5 of the MCMC algorithm. The full conditional posterior distribution of $\boldsymbol{\beta}$ is

$$\beta | \sigma_k^2, \boldsymbol{y} \sim N(\hat{\beta}, V_\beta \sigma_k^2).$$

The estimate $\hat{\beta}$ is used in step 2 of the MCMC algorithm, instead of the full distribution.

The posterior distribution of the parameter ψ_k is

$$p(\psi_k | \boldsymbol{y}) \propto p(\psi_k) |V_\beta|^{1/2} (s_k^2)^{-(n-q)/2}.$$

A natural choice for the prior distributions, $p(\psi_k)$, is the uniform $U([0,1])$.

Alternatively, as explained below, the parameters ψ_k, $k = 1, 2, \ldots, K$ can be computed by least squares estimation within the MCMC sampler. Let $e_k(t) = y_k(t) - \hat{M}_k(t)$ be the residuals at time t of experiment k. Based on our normality assumption, the residuals are

$$e_k(t) \sim N(0, \sigma_k^2 v_k(t)).$$

Thus,

$$Var\left(\left(\frac{e_k(t)}{\sigma_k}\right)\right) = E\left(\left(\frac{e_k(t)}{\sigma_k}\right)^2\right) = w_k(t)^{-\psi_k}$$

Assuming regularity conditions

$$E\left(\log\left(\left(\frac{e_k(t)}{\sigma_k}\right)^2\right)\right) = -\psi_k \log w_k(t)$$

and thus we can get ψ_k from the linear regression of

$$\log\left(\frac{e_k(t)}{\sigma_k}\right)^2 \text{ on } -\log w_k(t).$$

We would also like to note that the least square estimation of the parameters in β makes them deterministic functions of the rest of the TRS model parameters. Therefore, the term often referred as Jacobian that typically appears in the acceptance ratio of trans-dimensional moves is equal to 1 because the proposed parameters do not alter the value of the current parameter vector except from the parameters in β that, because they are deterministically derived from the rest of the current parameters, have a degenerate proposal distribution.

5 Simulation studies

We next provide details related to the simulation studies in Sect. **I**3.1 and **I**3.2. In both studies, the simulated data satisfy

$$Y(t) = M(t) + \sigma(t)Z, \qquad M(t) = \Omega h(t), \quad \sigma(t) = \sigma_1\sqrt{\Omega h(t)}, \quad Z \sim N(0,1), \tag{4}$$

where $\Omega = 1000$ and $\sigma_1 = 20$. The values of Ω and σ_1 are chosen so that the magnitude and variability of the simulation data is chosen to be similar to the real data examples considered in Sect. **I**4 while $\sigma(t)$ is set proportional to $\sqrt{h(t)}$ to allocate greater variability for higher levels of mRNA. Multiple hypothetical experiments are considered in both simulation studies. Two experiments are considered in the first simulation study and four in the second study as opposed to three experiments considered in the real data examples. As in the real data

example, four replications are generated for each experiment in both simulation studies.

The deterministic profiles, $h(t)$, for all genes in the network are produced as solutions of ODEs. For those genes that are regulated by some regulator in the network, the transcription rates are of the form of Hill-type functions. This is a standard approach used to produce flexible S-shaped transcription regulation functions (see for example Santillán [2008]; Bhaskaran et al. [2015]). For those transcription functions, the input to the Hill function is the nuclear concentration level of the TF protein associated with the regulating gene. This is derived using equations as in (S2).

The same values for the transcription, mRNA degradation, translation, protein degradation, import and export rates are used across experiments of each simulation study. The parameters of the Hill functions for the target transcription rate are also the same across experiments. The values of translation, protein degradation, import and export rates are also the same across regulators. The values of transcription and mRNA degradation rates as well as the initial values of each experiment are chosen to generate network dynamics. For example, in the first simulation study where the candidate f_6 has profile with similar form with the regulator f_1 but overall f_1 is more dynamic, the transcription and mRNA degradation rate of f_1 are larger than those of f_6. More details for each simulation study are given below.

5.1 Repressed activation network

The logic of the interaction in this simulation study is that a TF f_1 activates the target T in the absence of a repressor TF, f_2. When f_2 is present, the activation is repressed and prevented. The other candidate regulators, f_3, f_4, f_5 and f_6 are not involved in the interaction but they have profiles that, as we discussed in Sect. **I**3.1, can provide alternative options to the regulation set $\Phi = \{f_1, f_2\}$. Figure 3 provides a logic diagram of this network.

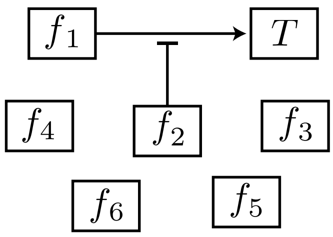

Figure 3: **Logic diagram of the artificial repressed activation network.**

The profiles, $h_i(t)$, where $i = 1, 2, \dots, 6$ and T (target gene) using which we generate the artificial data $Y_i(t)$ as in (4) for each gene in the network are derived as solutions of the following ODE system.

$$\begin{aligned}
\dot{h}_1 &= \tau_1(t) - \delta_1 h_1(t), \quad \tau_1(t) = \tau_1 g_1(t), \\
\dot{h}_2 &= \tau_2(t) - \delta_2 h_2(t), \quad \tau_2(t) = \tau_2 g_2(t), \\
\dot{h}_3 &= \tau_3(t) - \delta_3 h_3(t), \quad \tau_3(t) = \tau_3 g_3(t), \\
\dot{h}_4 &= \tau_4(t) - \delta_4 h_4(t), \quad \tau_4(t) = \tau_4 g_4(t), \\
\dot{h}_5 &= \tau_5(t) - \delta_5 h_5(t), \quad \tau_5(t) = \tau_5 g_5(t), \\
\dot{h}_6 &= \tau_6(t) - \delta_6 h_6(t), \quad \tau_6(t) = \tau_6 g_6(t), \\
\dot{Pc}_1 &= \alpha h_1(t) - \delta_P Pc_1(t) - k_i Pc_1(t) + k_e Pn_1(t), \\
\dot{Pn}_1 &= k_i Pc_1(t) - k_e Pn_1(t) - \delta_P Pn_1(t), \\
\dot{Pc}_2 &= \alpha h_2(t) - \delta_P Pc_2(t) - k_i Pc_2(t) + k_e Pn_2(t), \\
\dot{Pn}_2 &= k_i Pc_2(t) - k_e Pn_2(t) - \delta_P Pn_2(t), \\
\dot{h}_T &= \tau_3 \frac{Pn_1(t)^h}{Pn_1(t)^h + k^h} \frac{k^h}{Pn_2(t)^h + k^h} - \delta_T h_T(t)
\end{aligned}$$

As we explained in the previous section, the transcription regulation of the target gene by regulators f_1 and f_2 is modelled by Hill functions. The values of the parameters used for the Hill functions are $k = 5$ and $h = 10$. These give the S-shaped transcription functions in Fig. S4.

The values of the protein translation, degradation, import and export rates are $\alpha = \log(2)/0.5$, $\delta_P = \log(2)/2$, $k_i = 2$, $k_e = 1$, respectively. The values of the mRNA degradation rates are $\delta_1 = \delta_2 = \delta_3 = \delta_T = \log(2)/1.25$, $\delta_4 = \delta_6 = \log(2)/2$ and $\delta_5 = \log(2)/0.5$. These give half-life between $0.5 - 2h$. The different values are due to the various different profiles that we wish to give to the alternative candidate regulators f_3, f_4, f_5 and f_6 compared to genes that are part of the repressed activation network. More specifically, the regulator f_4 has slower degradation to provide a profile that remains constant at low or high levels in the

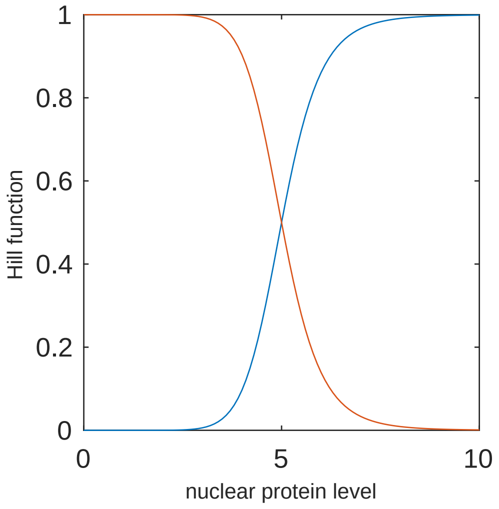

Figure 4: Hill-type functions of the form $Pn^h/(Pn^h+k^h)$ for activation (blue) and $k^h/(Pn^h+k^h)$ for repression (red) as a function of the nuclear protein levels Pn for parameters $k = 5$ and $h = 10$.

first and second experiment, respectively. The regulator f_5 has larger degradation to provide a profile similar to a reflection of regulator f_2, which degrades quickly (f_2 transcripts quickly). Finally, the regulator f_6 has slower degradation as this provides similar form with f_1 but with slower and smaller scale changes.

The transcription rate functions $\tau_i(t)$, $i = 1, 2, \ldots, 6$ are of the form $\tau_i g_i(t)$ where $\tau_1 = \tau_2 = \tau_3 = \tau_5 = \tau_T = \log(2)/0.25$, $\tau_4 = \log(2)/0.5$ and $\tau_6 = \log(2)/0.75$. The values of τ_i's differ across genes similarly to the values of the degradation rates. The regulator f_4 and f_6 have slower transcription rates because the former remains fairly constant in both experiments and the latter, f_6, changes slower than f_1. The functions $g_i(t)$ provide the form of the profiles. As previously discussed, we wish that the regulators f_1 and f_6 have the same profile and thus in the first experiment $g_1(t) = g_6(t) = 1$, $t \in [0, 9)$ and $g_1(t) = g_6(t) = 0$, $t \in [9, 10]$ and in the second experiment $g_1(t) = g_6(t) = 1$, $t \in [0, 1)$ and $g_1(t) = g_6(t) = 0$, $t \in [1, 10]$. This provides the activation of the target gene in the first experiment, while in the second experiment the form of $g_1(t)$ implies that the regulator quickly starts to degrade causing a fall of the target transcription rate to basal levels at about the middle of the observed time interval. We also wish that the regulator f_3 has the same profile in both experiments and this is the same with the profile of f_2 in experiment 1. Thus, in both experiments $g_3(t) = 0$, $t \in [0, 3)$ and $g_3(t) = 1$, $t \in [3, 10]$. The regulator f_2 has the same profile with f_3 in the first experiment

and $g_2(t) = 1$, $t \in [0, 10]$ in the second experiment. This produce repressions of the target in both experiments with the repression occurring earlier in the second experiment. To produce a profile constantly at low levels in the first experiment for f_4, $g_4(t) = 0.1$, $t \in [0, 10]$, while in the second experiment $g_4(t) = 1$, $t \in [0, 10]$ that produces a profile constantly at high levels (in combination with a high initial value, see below). For the fifth candidate, f_5, which has a profile similar to a reflection of the profile of f_2, the profile in the first experiment is $g_5(t) = 1$, for $t \in [0, 5)$ and $g_5(t) = 0$, for $t \in [5, 10]$. In the second experiment the profile of f_5 is switched off for the whole observed time-interval, $g_5(t) = 0$, $t \in [0, 10]$.

The initial conditions are also set appropriately to produce these profiles. More specifically, in the first experiment $h_1(0) = h_2(0) = h_3(0) = 1$, $h_T(0) = h_4(0) = h_6(0) = 2$, $h_5(0) = 7$, $Pc_1(0) = Pc_2(0) = Pn_1(0) = Pn_2(0) = 1$. In the second experiment, $h_1(0) = h_4(0) = 6$, $h_2(0) = 2$, $h_T(0) = h_3(0) = 1$, $h_5(0) = 7$, $h_6(0) = 4$ and $Pc_1(0) = Pn_1(0) = 6$, $Pc_2(0) = Pn_2(0) = 2$.

A MATLAB script file that contains all the above parameter specifications is provided with the TRS software.

5.1.1 Simulated data

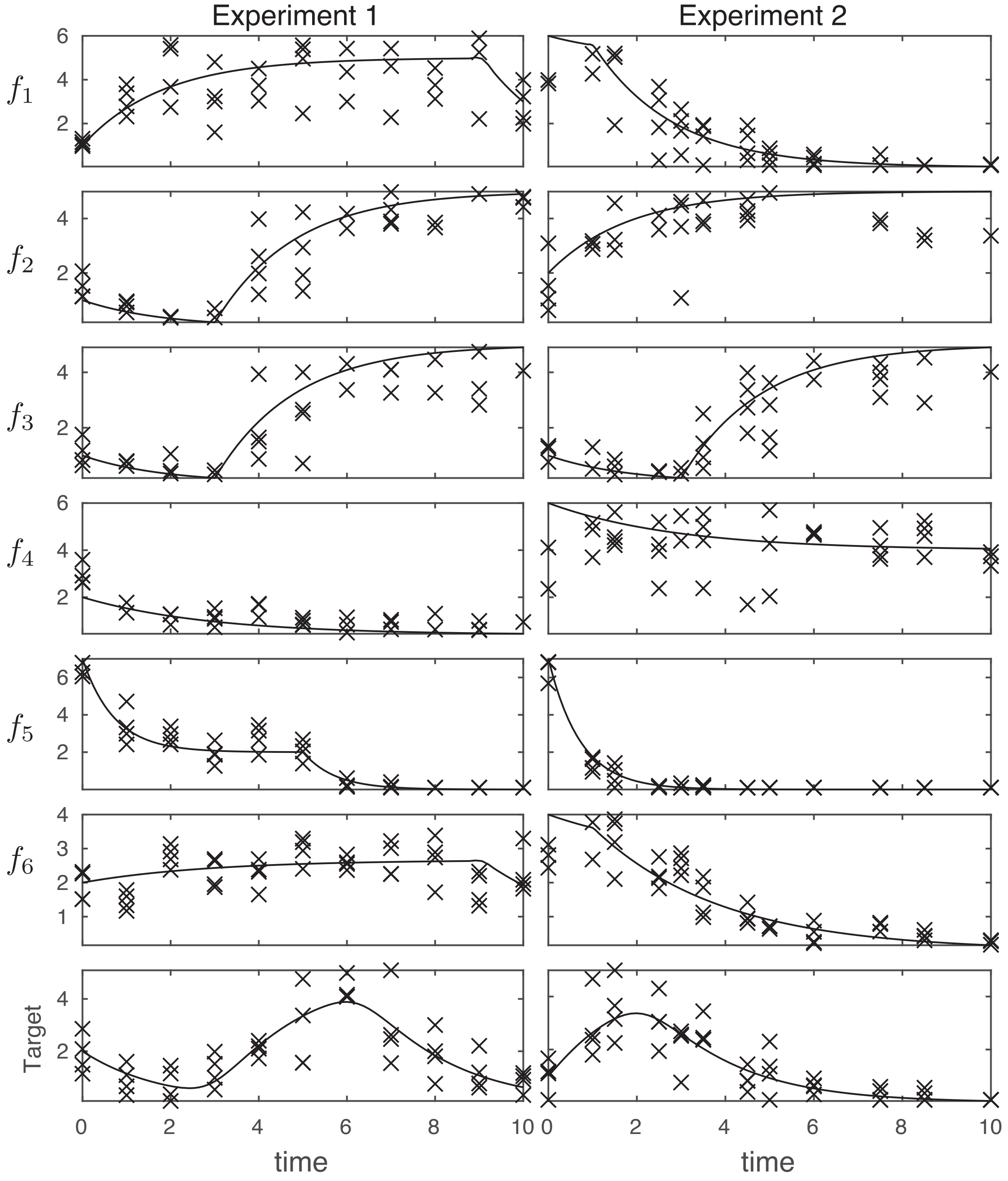

Figure 5: The pseudo-random data (crosses) generated for the simulation study and their underlying deterministic profiles (solid lines).

5.1.2 Prior distributions

The prior distribution used for the results reported in Sect. **I**3.1.

- for the degradation rate, δ, is a Gamma distribution with mean 0.3450 and standard

deviation 0.1543,

- for the precision σ_k^{-2}, $k = 1, 2$, is a scaled χ^2 distribution with $n_{k,0} = 0.001$ degrees of freedom and scale $\sigma^2_{k,0} = 0.001$

- for the number of regulators, a Poisson prior with parameter $\lambda = 0.15$

- for the choice of regulators, $\pi(f \in \Phi) = 0.1627, 0.1925, 0.2585, 0.2264, 0.0569, 0.0833, 0.0196$ for $f = f_1, \ldots, f_6, T$, respectively.

- for the threshold priors the cut-off value $\bar{g}_{min}$ is set equal to the median of the standardized gradient values $\bar{g}_f^{(k)}(t)$ across the observed time-intervals of all experiments.

The prior distributions for the number of regulators, for the choice of regulators and for the threshold priors used in the results discussed in Sect. **I**3.1 are presented in Fig. S6.

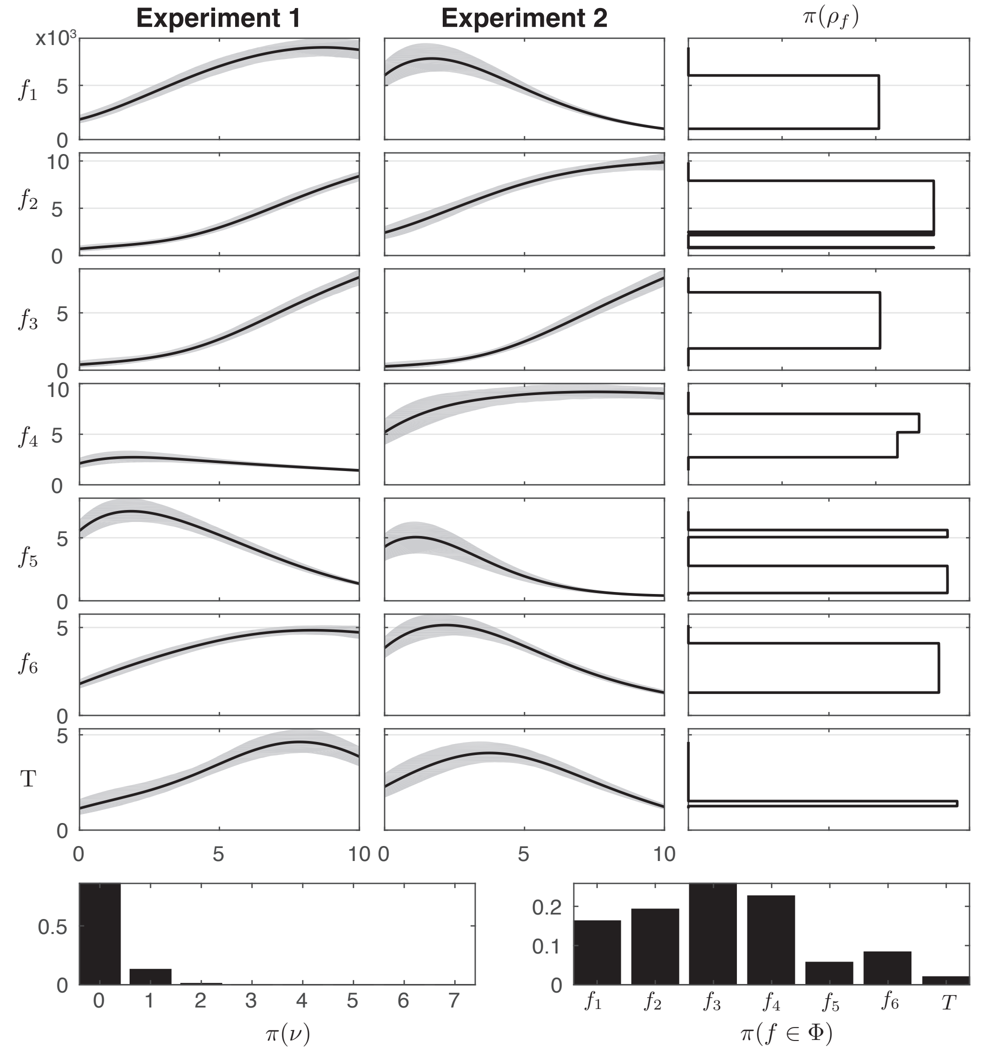

Figure 6: **Prior distributions for the number of regulators $\pi(\nu)$(bottom left), the probability of each candidate, f, $f = f_1, f_2, \ldots, f_6, T$ to be in the regulators' set $\pi(f \in \Phi)$ (bottom right) and for the threshold value of each regulator, $\pi(\rho_f)$ (right panel, rows 1-7).** The left and centre panel of rows 1-7 provide the smoothed regulators profiles, $P_f^{(k)}(t)$, $k = 1, 2$ (solid line) and their 90% bootstrap confidence envelope (grey area) in the first and second experiment, respectively. All the plots in each of rows 1-7 correspond to the same regulator and they have the same y-axis. The hyperparameters for all the displayed prior distributions are provided in the section above. These priors are used for deriving the results of the simulation study discussed in Sect. **I**3.1.

5.1.3 Monte Carlo Markov Chains of various parameters

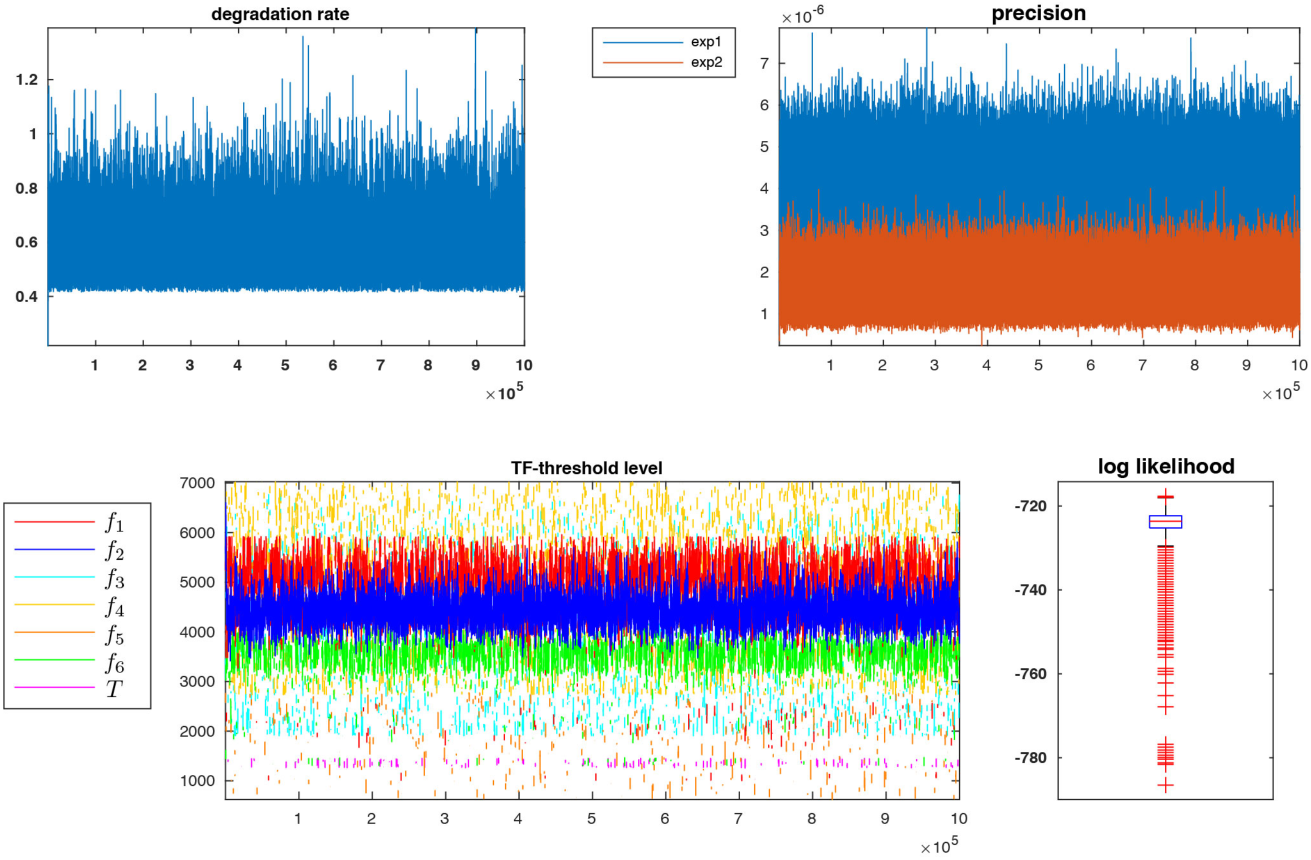

Figure 7: Monte Carlo Markov Chains of the parameters degradation rate (δ), precision (σ_k^{-2}) and threshold level (ρ_f, $f = f_1, f_2, \ldots, f_6, T$) of the TRS model for the simulation example discussed in Sect. **I**3.1.

We also provide the running averages of various parameters, namely the degradation rate, the precision in each experiment and the threshold levels during the MCMC run as well as the running estimate of the probability of the number of selected TFs. This clearly show the quick convergence of these parameters during the MCMC run.

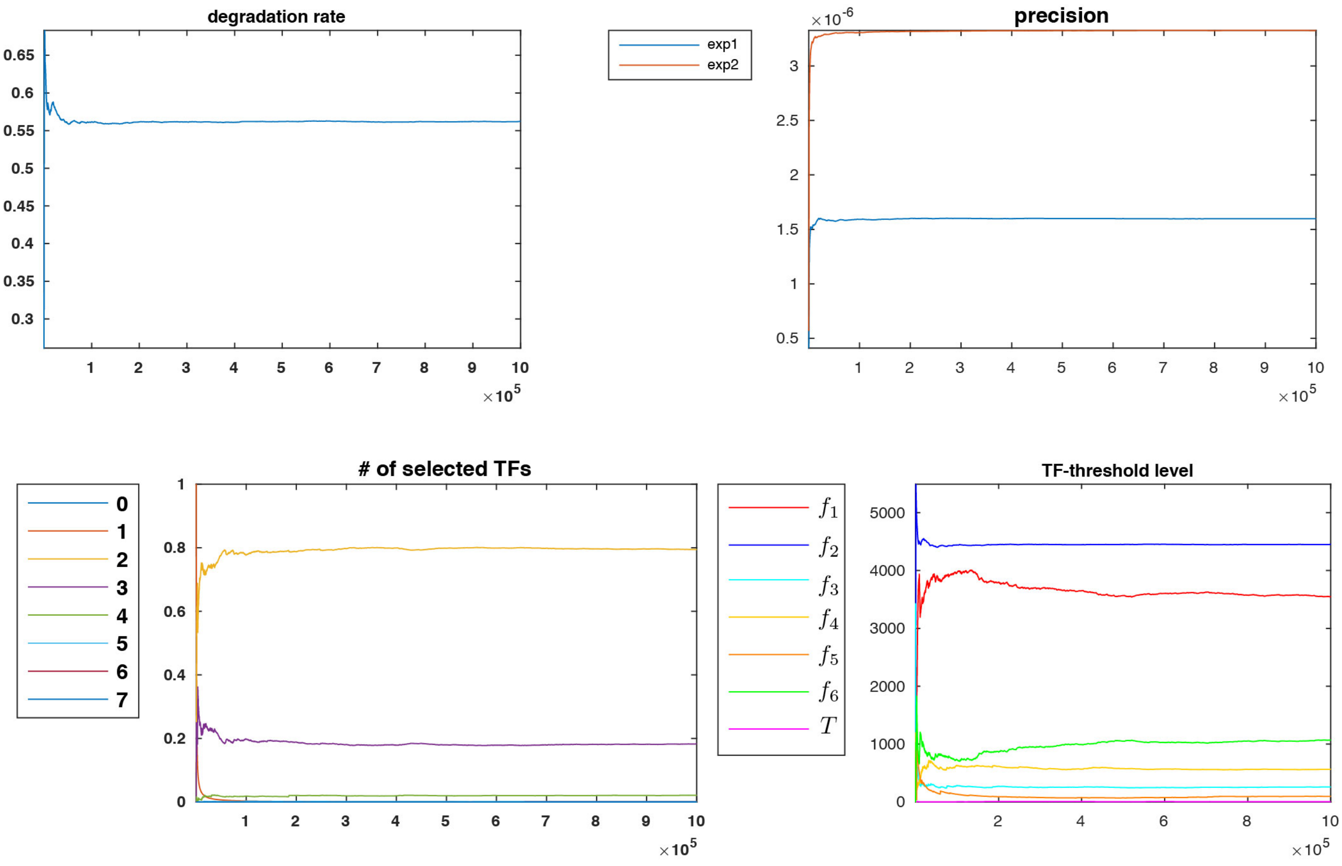

Figure 8: Monte Carlo Markov Chains of the parameters degradation rate (δ), precision (σ_k^{-2}) and threshold level (ρ_f, $f = f_1, f_2, \ldots, f_6, T$) of the TRS model for the simulation example discussed in Sect. **I**3.1.

5.1.4 Inference

Fig. 9 provides a summary of the results discussed in Sect. **I**3.1.

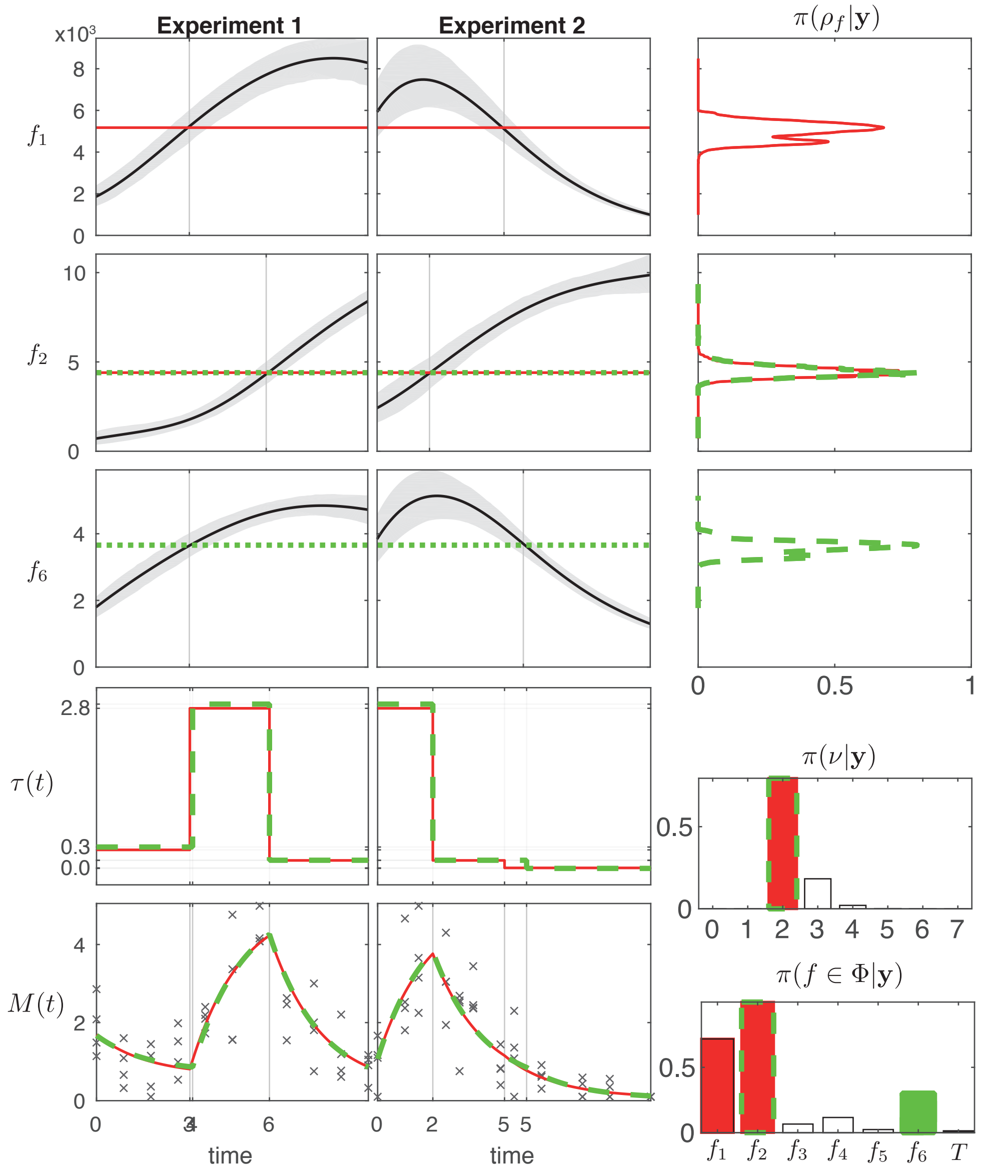

Figure 9: **Posterior inference for the simulation study of the repressed activation network using the TRS model.** This is a summary figure for the results discussed in Sect. **I**3.1. The posterior probabilities of having ν regulators (see right, 2nd bottom panel), of selecting each of the candidate regulator (right, bottom panel) and for their thresholds (right, top 3 panels) are presented. The profiles of the three most likely regulators are presented in left and centre panel of rows 1-3 along with the mode of the thresholds' posterior distributions. All plots in each of rows 1-3 have the same y-axis. The two *a posteriori* most likely models are marked with red and green color. The transcription profiles and fit to target data of these two models are respectively displayed in the left and centre panel of the two bottom rows.

5.1.5 Results for less informative prior distributions

In this section we report results of TRS algorithm for two different sets of prior distributions.

Both of these set of prior distributions use the same priors for the degradation rate and

precision parameters as in Sect. S5.1.2. The first set of these prior distributions also has the same Poisson($\lambda = 0.15$) prior for the number of regulators as in Sect. S5.1.2, but uses uniform distributions both for the choice of regulators (discrete uniform) and the threshold of the regulators (uniform in the covered range of each regulator). The second set of these prior distribution uses a larger $\lambda = 1$ parameter for the number of regulators with all other priors being the same as the first set. Fig. S10 present the Monte Carlo Markov Chains a resulted from a run of 1M iterations of the TRS algorithm using the first set of prior distributions. Fig. S11 provides a summary of the results from this implementation of TRS. Note the lower posterior probability for selecting f_1 compared to the case of informative priors (see Fig. S9) and larger posterior probability of selecting its alternative regulator f_6. This is due to that the priors in Fig. S9 give larger preference to the more dynamic profile of f_1.

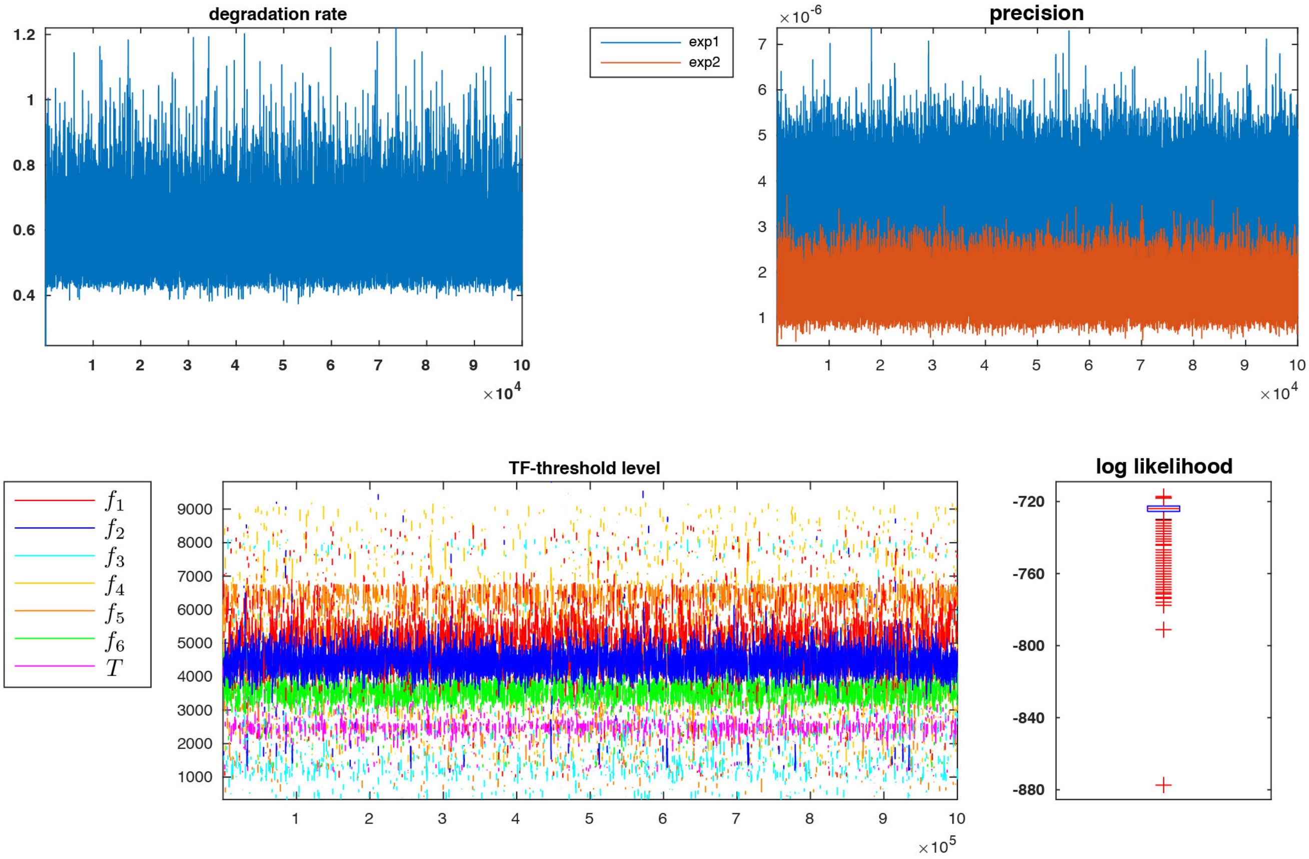

Figure 10: Monte Carlo Markov Chains of the parameters degradation rate (δ), precision (σ_k^{-2}) and threshold level (ρ_{f_j}, $j = 1, 2, \ldots, 15$) of the TRS algorithm for the simulation example of repressed activation network with less informative priors for the regulator set and their thresholds as explained above.

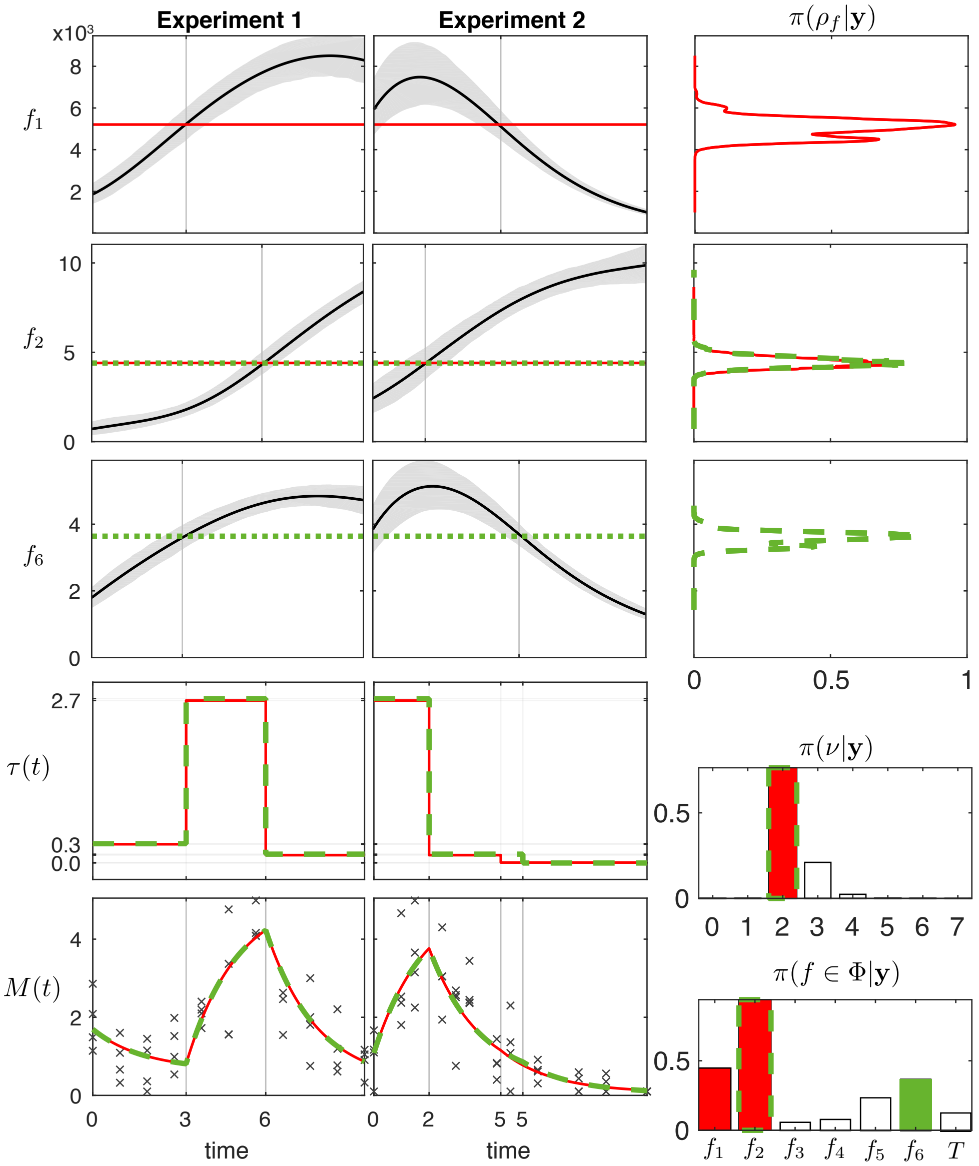

Figure 11: **Posterior inference for the simulation study of the repressed activation network using the TRS model.** Here less informative priors for the regulator set and their thresholds are used as explained above. The setup of the figure is the same as Fig. S9.

Fig. S12 present the Monte Carlo Markov Chains a resulted from a 1M iteration run of the TRS algorithm using the second set of less informative prior distributions. Fig. S13 provides a summary of the results from this implementation of TRS.

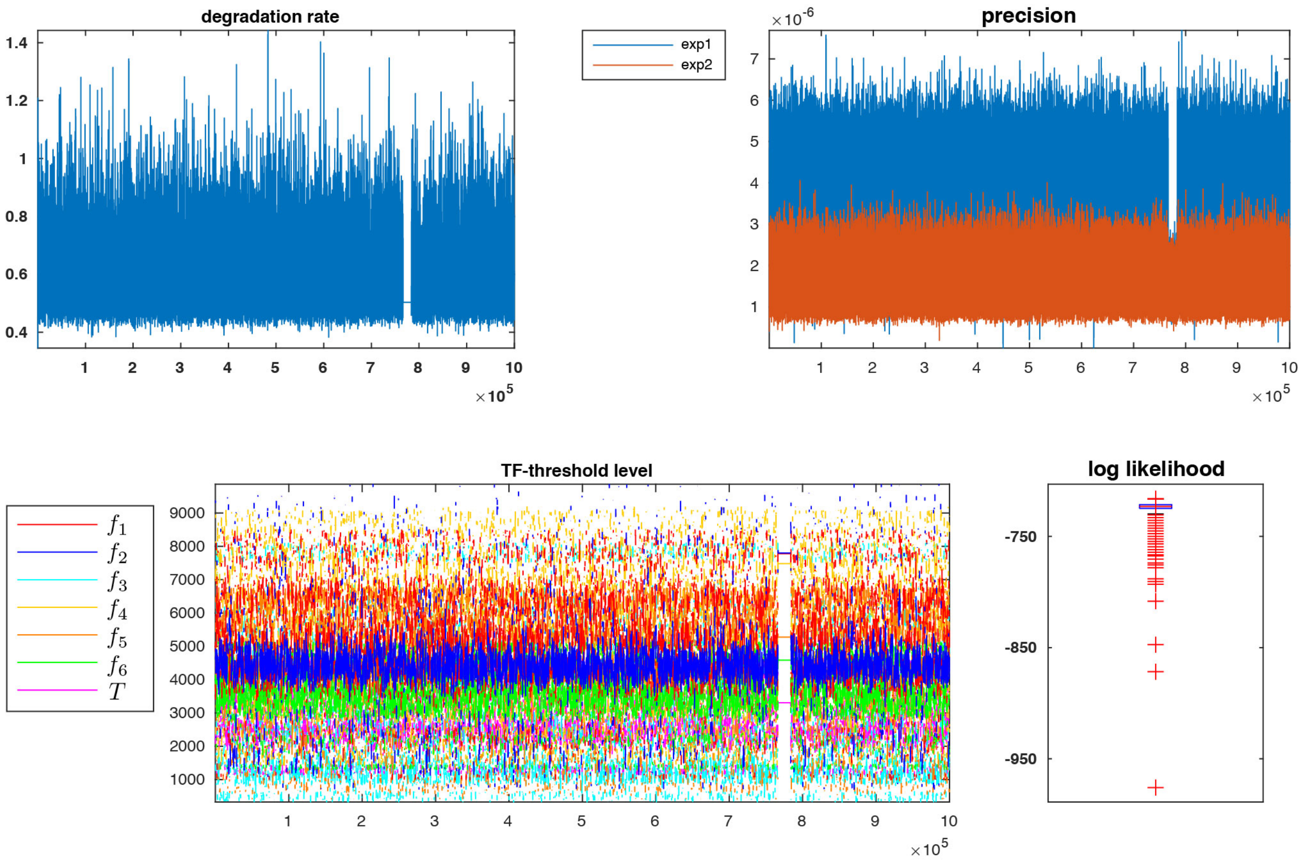

Figure 12: Monte Carlo Markov Chains of the parameters degradation rate (δ), precision (σ_k^{-2}) and threshold level (ρ_{f_j}, $j = 1, 2, \ldots, 15$) of the TRS algorithm for the simulation example of repressed activation network with less informative priors for the regulator set and their thresholds as explained above and Poisson($\lambda = 1$) prior for the number of regulators.

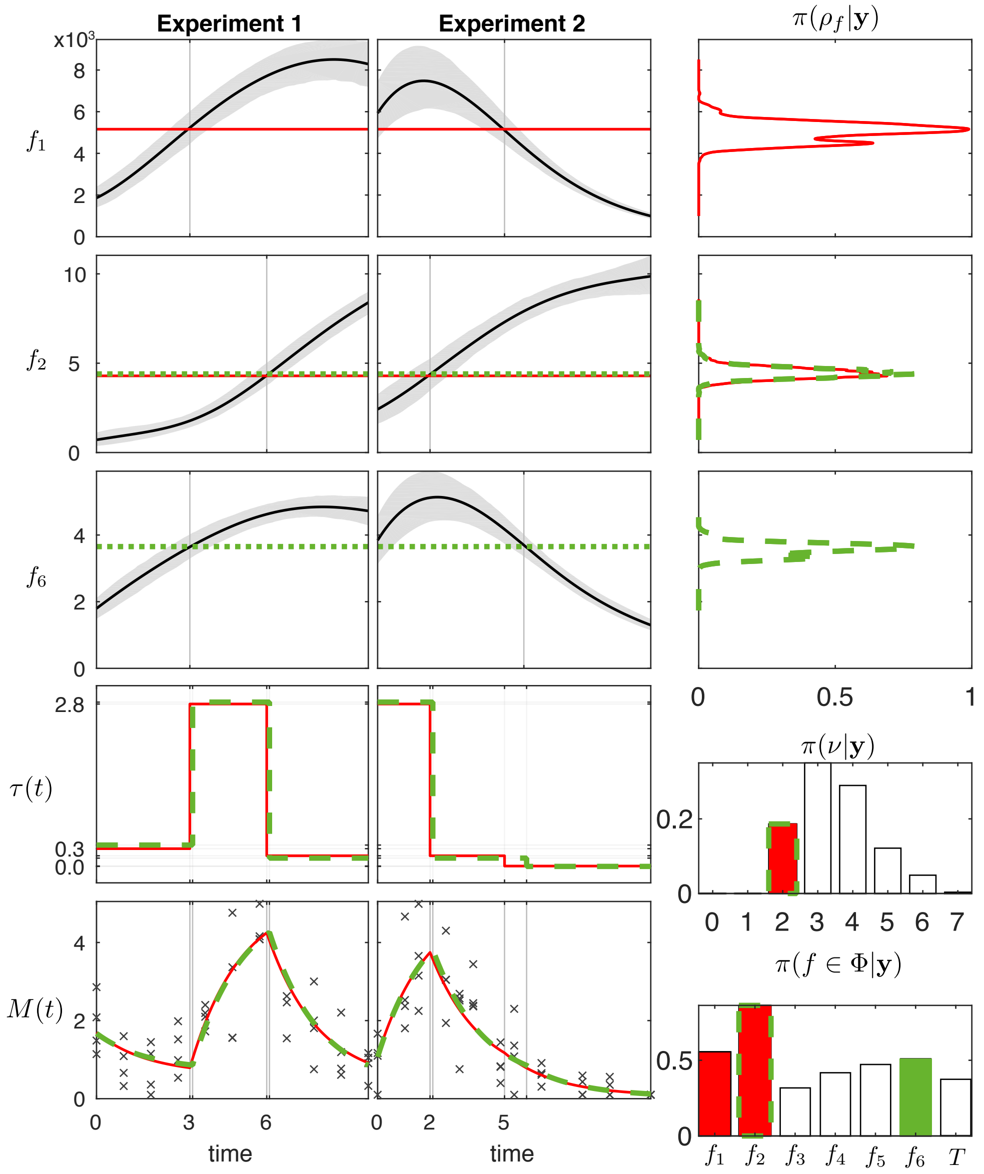

Figure 13: **Posterior inference for the simulation study of the repressed activation network using the TRS model.** Here less informative priors for the regulator set and their thresholds as explained above and Poisson($\lambda = 1$) prior for the number of regulators. The setup of the figure is the same as Fig. S9.

5.1.6 Results for smaller values of smoothing parameter

Following the discussion for the stability of the TRS algorithm to the smoothing splines in Sect. S2, we present the results of running the TRS algorithm with a smaller value of the smoothing parameters. This implies a better fit to the observed mRNA expression levels of the candidate regulators but less smooth regulator profiles. The same set of hyperparameters for the informative prior distributions is used as in the results in Sect. **I**3.1. However, the

change in the splines slightly affects the prior distributions for the choice of regulators and their thresholds (see Fig. S14). This is reflected with small changes in the results of the TRS algorithm (see Fig. S15 and S16). Note that the prior distributions are very similar to those in Fig. S6 and the results are very similar to those in Fig. S7 and S9.

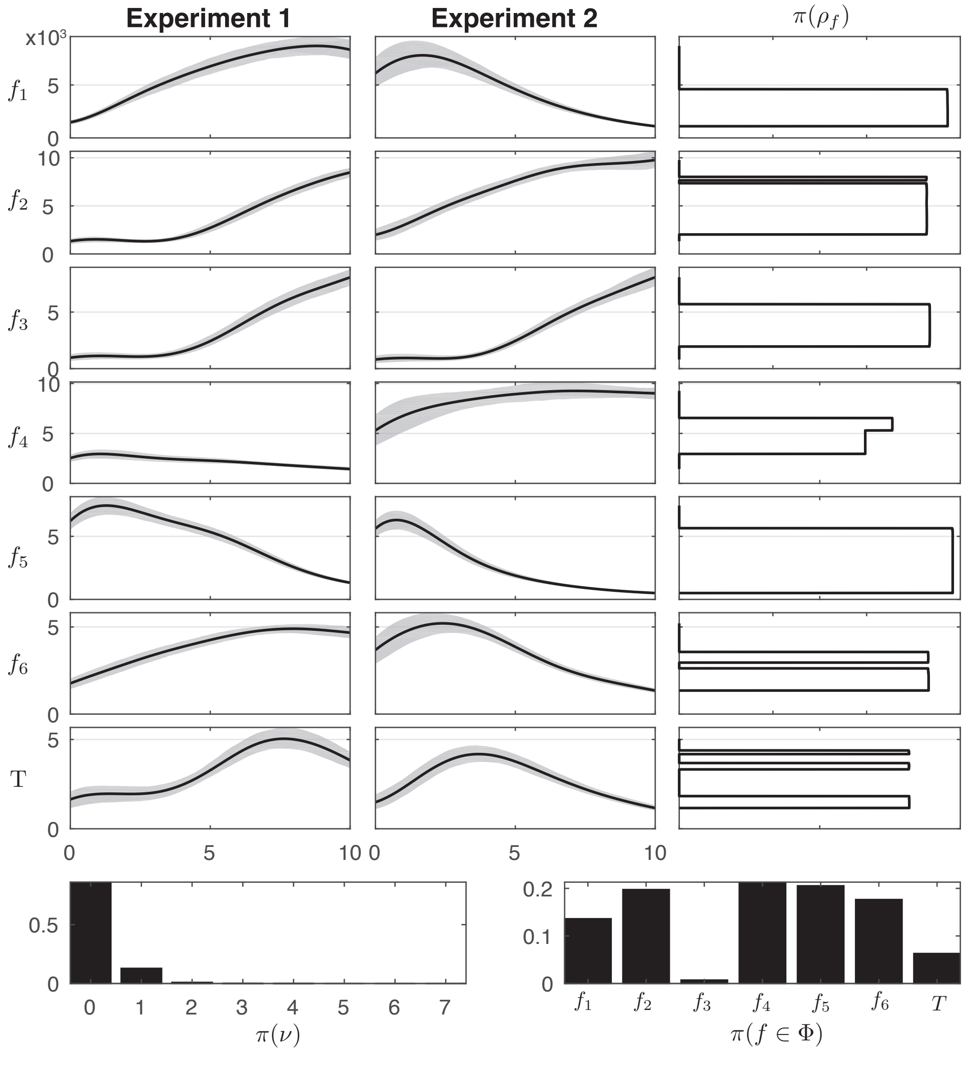

Figure 14: **Prior distributions for the number of regulators $\pi(\nu)$(bottom left) the probability of each candidate, f, $f = f_1, f_2, \ldots, f_6, T$ to be in the regulators set $\pi(f \in \Phi)$ (bottom right) and for the threshold value of each regulator, $\pi(\rho_f)$ (right panel, rows 1-7).** The left and centre panel of rows 1-7 provide the smoothed regulators profiles, $P_f^{(k)}(t)$, $k = 1, 2$ (solid line) and their 90% bootstrap confidence envelope (grey area) in the first and second experiment, respectively. All the plots in each of rows 1-7 correspond to the same regulator and they have the same y-axis. The hyperparameters for all the displayed prior distributions are the same as in section 5.1.2. The only difference is that the value of the smoothing parameter is smaller (0.5 as opposed to 0.9) which makes the spline estimates of the mRNA profiles of the candidate regulators less smooth but better fit to the mRNA expression data.

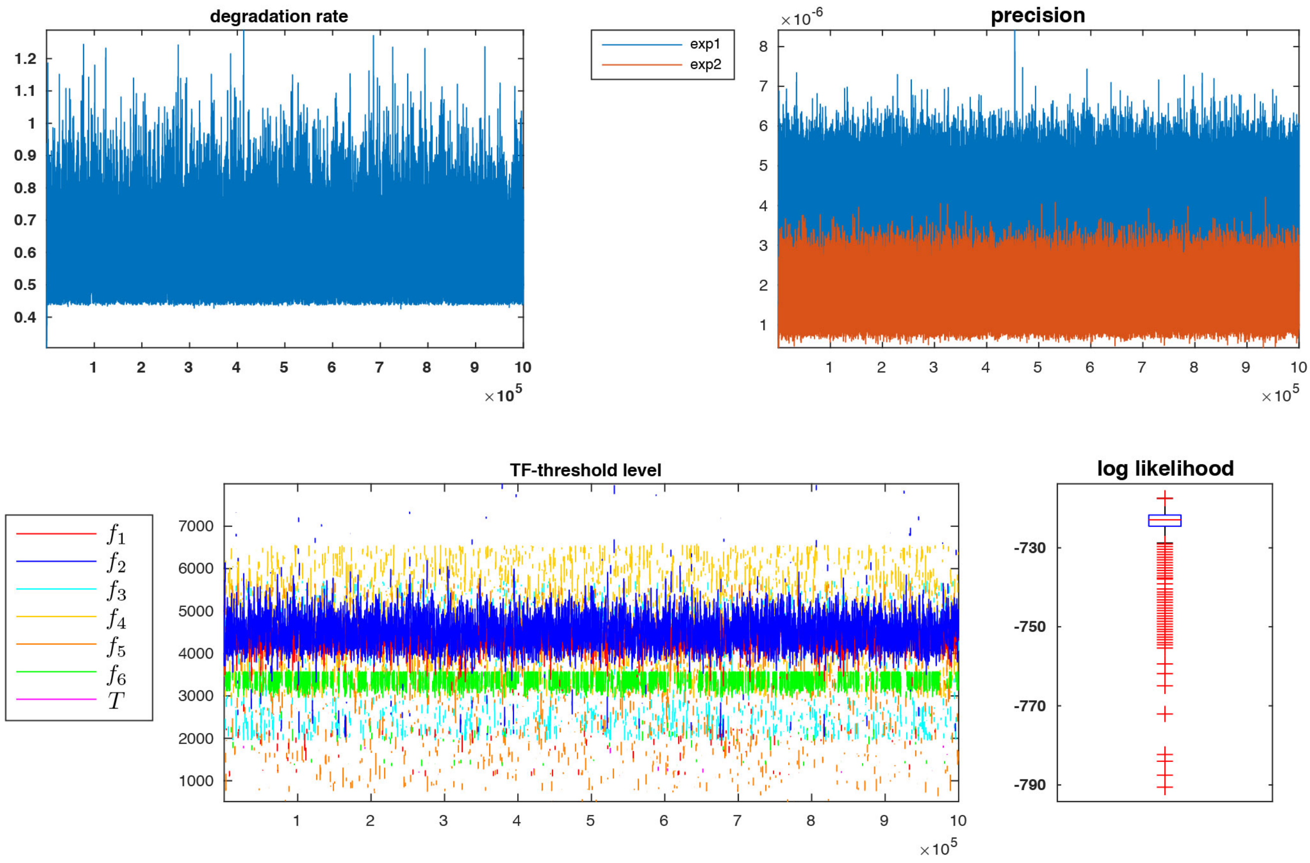

Figure 15: Monte Carlo Markov Chains of the parameters degradation rate (δ), precision (σ_k^{-2}) and threshold level ($\rho_f,\ f = f_1, f_2, \ldots, f_6, T$) of the TRS model for the prior distributions displayed in Fig. S14.

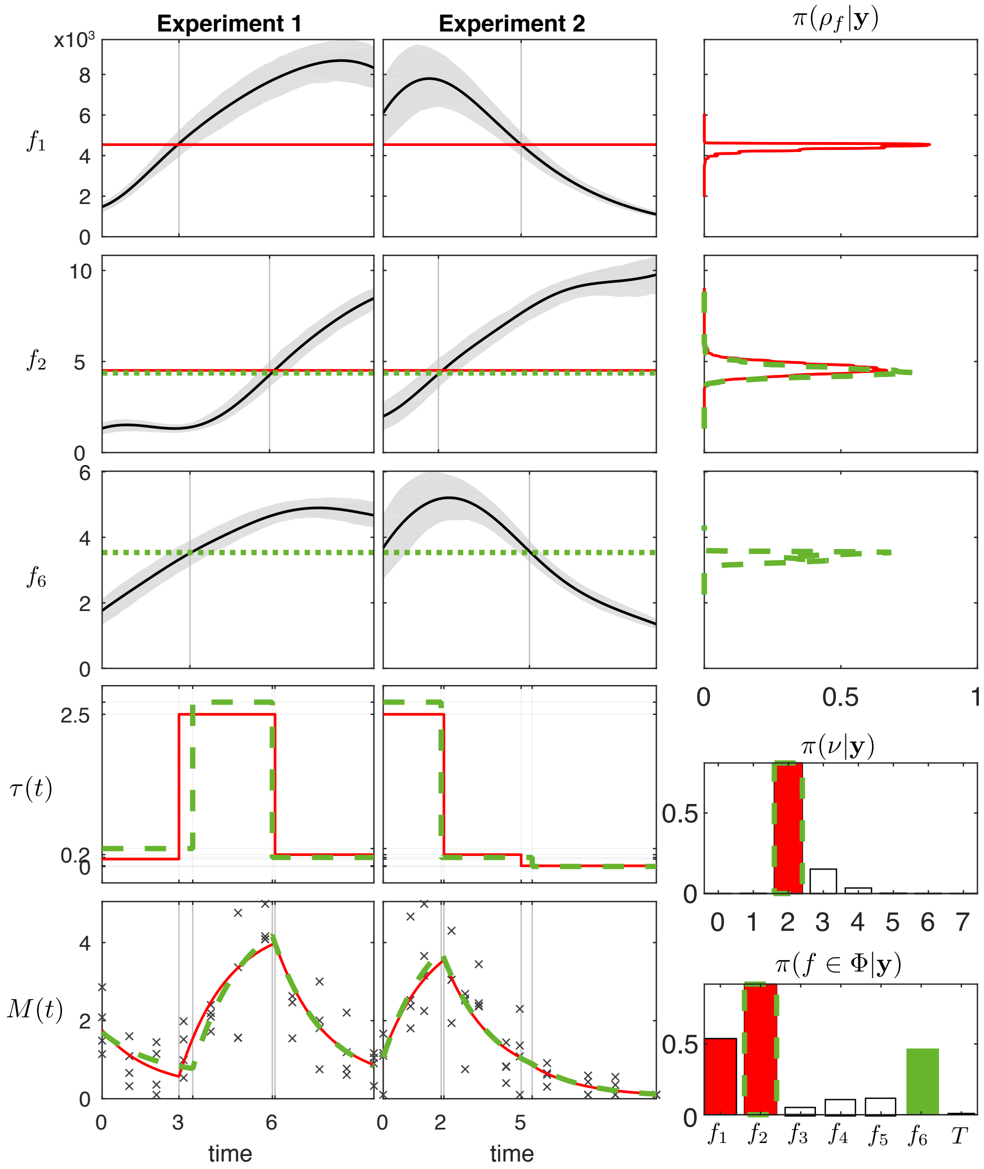

Figure 16: **Posterior inference for the simulation study of the repressed activation network using the TRS model with prior distributions as in Fig. 14**. The setup of the figure is the same as Fig. S9.

5.1.7 Output of the GRNInfer tool

Table 3 provides the output of the GRNInfer tool.

	f_1	f_2	f_3	f_4	f_5	f_6	T
f_1	-1.1732	-0.0314	-0.0269	0.0258	0.5697	0.5044	0.3537
f_2	0.0770	-0.5756	0.8423	0.2165	-0.2925	0.9070	-0.0372
f_3	-0.0035	0.1969	-0.3259	0.0116	-0.0050	-0.0320	-0.1510
f_4	-0.3142	0.2941	0.0861	-0.8983	0.9419	-0.7216	2.3870
f_5	-0.0020	0.0033	-0.0314	0.0038	-0.9825	0.0380	0.0304
f_6	-0.0504	0.0141	-0.0035	0.0045	0.1597	-0.7511	0.0953
T	0.0333	0.0213	-0.0072	0.0069	-0.0424	0.8522	-1.2955

Table 3: The output of the GRNInfer tool for the data produced in this simulation study. The (i, j) entry of the table corresponds to the coefficient of the regulation of the i-th gene by the j-th gene where the names of the regulating and target genes are given in the first row and first column, respectively.

5.2 Flowering time network

Here we consider the gene regulation network related to the flowering time of *A. thaliana* published in Leal Valentim et al. [2015]. In particular, we focus on the regulation of the target gene SUPPRESSOR OF OVEREXPRESSION OF CONSTANS (SOC1). The latter gene has five regulators in this network: two repressors and three activators. The gene SOC1 can be activated by FLOWERING LOCUS T (FT). Another activator is SOC1 which implies that the target is autoregulated. The other activator is AGAMOUS-LIKE 24 (AGL24) for which SOC1 is also an activator. The two repressors, FLOWERING LOCUS C (FLC) and SHORT VEGETATIVE PHASE (SVP), and the activator FT are not regulated by species in the network. Finally, there are 3 more other genes, namely FD, LEAFY (LFY), APETALA 1 (AP1), in this network. Fig. 17 provides a diagram of the regulation.

Next, we provide an ODE system that represents this regulation. As in the previous simulation study, regulation of the target gene (either activation or repression) is modelled by Hill functions where the input is the nuclear concentration of the regulator protein associated with the regulating gene. The solutions, $h_i(t)$, $i = 1, 2, \ldots, 8$, provide the profiles of the genes in the network. As we explained above, these profiles are then used to generate data that satisfy equation (S4).

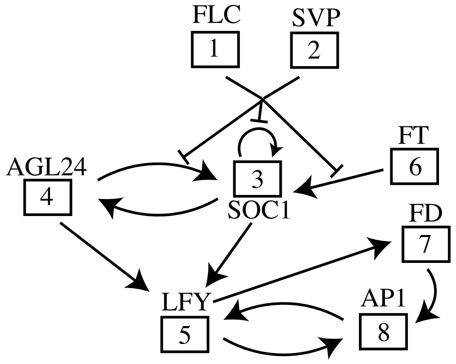

Figure 17: Diagram of the logic of the flowering time system of *A. thaliana*.

$$
\begin{aligned}
\dot{h}_1 &= \tau_1(t) - \delta_1 h_1(t), \quad \tau_1(t) = \tau_1 g_1(t), \\
\dot{Pc}_1 &= \alpha h_1(t) - \delta_P Pc_1(t) - k_i Pc_1(t) + k_e Pn_1(t), \\
\dot{Pn}_1 &= k_i Pc_1(t) - k_e Pn_1(t) - \delta_P Pn_1(t), \\
\dot{h}_2 &= \tau_2(t) - \delta_2 h_2(t), \quad \tau_2(t) = \tau_2 g_2(t), \\
\dot{Pc}_2 &= \alpha h_2(t) - \delta_P Pc_2(t) - k_i Pc_2(t) + k_e Pn_2(t), \\
\dot{Pn}_2 &= k_i Pc_2(t) - k_e Pn_2(t) - \delta_P Pn_2(t), \\
\dot{h}_3 &= \tau_3 \left(2\frac{Pn_3(t)^h}{Pc_3(t)^h + k_{33}^h} + \frac{Pn_4(t)^h}{Pn_4(t)^h + k_{43}^h} + \frac{Pn_6(t)^h}{Pn_6(t)^h + k_{63}^h} \right) \frac{k_{13}^h}{Pn_1(t)^h + k_{13}^h} \frac{k_{23}^h}{Pn_2(t)^h + k_{23}^h} - \delta_3 h_3(t) \\
\dot{Pc}_3 &= \alpha h_3(t) - \delta_P Pc_3(t) - k_i Pc_3(t) + k_e Pn_3(t), \\
\dot{Pn}_3 &= k_i Pc_3(t) - k_e Pn_3(t) - \delta_P Pn_3(t), \\
\dot{h}_4 &= \tau_4 \frac{Pn_3(t)^h}{Pn_3(t)^h + k_{34}^h} - \delta_4 h_4(t) \\
\dot{Pc}_4 &= \alpha h_4(t) - \delta_P Pc_4(t) - k_i Pc_4(t) + k_e Pn_4(t), \\
\dot{Pn}_4 &= k_i Pc_4(t) - k_e Pn_4(t) - \delta_P Pn_4(t), \\
\dot{h}_5 &= \tau_5 \left(\frac{Pn_3(t)^h}{Pn_3(t)^h + k_{35}^h} + \frac{Pn_4(t)^h}{Pn_4(t)^h + k_{45}^h} + \frac{Pn_8(t)^h}{Pn_8(t)^h + k_{85}^h} \right) - \delta_5 h_5(t) \\
\dot{Pc}_5 &= \alpha h_5(t) - \delta_P Pc_5(t) - k_i Pc_5(t) + k_e Pn_5(t), \\
\dot{Pn}_5 &= k_i Pc_5(t) - k_e Pn_5(t) - \delta_P Pn_5(t), \\
\dot{h}_6 &= \tau_6(t) - \delta_6 h_6(t), \quad \tau_6(t) = \tau_6 g_6(t), \\
\dot{Pc}_6 &= \alpha h_6(t) - \delta_P Pc_6(t) - k_i Pc_6(t) + k_e Pn_6(t), \\
\dot{Pn}_6 &= k_i Pc_6(t) - k_e Pn_6(t) - \delta_P Pn_6(t), \\
\dot{h}_7 &= \tau_7 \frac{Pn_5(t)^h}{Pn_5(t)^h + k_{57}^h} - \delta_7 h_7(t) \\
\dot{Pc}_7 &= \alpha h_7(t) - \delta_P Pc_7(t) - k_i Pc_7(t) + k_e Pn_7(t), \\
\dot{Pn}_7 &= k_i Pc_7(t) - k_e Pn_7(t) - \delta_P Pn_7(t), \\
\dot{h}_8 &= \tau_8 \left(\frac{Pn_5(t)^h}{Pn_5(t)^h + k_{58}^h} + \frac{Pn_7(t)^h}{Pn_7(t)^h + k_{78}^h} \right) - \delta_8 h_8(t) \\
\dot{Pc}_8 &= \alpha h_8(t) - \delta_P Pc_8(t) - k_i Pc_8(t) + k_e Pn_8(t), \\
\dot{Pn}_8 &= k_i Pc_8(t) - k_e Pn_8(t) - \delta_P Pn_8(t),
\end{aligned}
$$

Pseudo-random data are generated for four hypothetical experiments. Similarly to the previous simulation study, the four experiments differ in terms of the profiles $g_i(t)$, where $\tau_i(t) = \tau_i g_i(t)$, $i = 1, 2, 6$ and the initial conditions. All the other parameters are the same in all experiments. For each experiment, four replicates are generated with exactly the same parameters.

As in the previous simulation study, the values of the protein translation, degradation, import and export rates are $\alpha = \log(2)/0.5$, $\delta_P = \log(2)/2$, $k_i = 2$, $k_e = 1$ for all 8 genes. Here, the values of the transcription and mRNA degradation rates are also the same for all genes, $\delta_i = \ln(2)/1.5$, $\tau_i = \ln(2)/0.25$, $i = 1, 2, \ldots, 8$.

The values of the Hill parameters are $k_{13} = k_{23} = k_{63} = 5$, $k_{34} = k_{43} = k_{35} = k_{4.5} = k_{57} = k_{78} = k_{58} = k_{85} = 7.5$, $k_{33} = 10$ and $h = 10$. Fig. 4 provides an example of such type of transcription rate functions.

The profiles $g_i(t)$, $i = 1, 2, 6$ and the initial conditions listed next are chosen to generate the various dynamic profiles in different hypothetical experiments that are presented in Fig. S18. For the first experiment,

$$g_1(t) = g_2(t) = 0.1, \quad t \in [0, 10], \qquad g_6(t) = 0, \quad t \in [0, 2), \quad g_6(t) = 1, \quad t \in [2, 10],$$

with initial conditions

$$h_1(0) = h_2(0) = h_4(0) = h_6(0) = h_8(0) = 1, \quad h_3(0) = h_5(0) = h_7(0) = 2.$$

For the second experiment,

$$g_1(t) = 0.1, \ t \in [0, 10], \quad g_2(t) = 0, \ t \in [0, 5), \ g_2(t) = 1, \ t \in [5, 10],$$
$$g_6(t) = 0, \ t \in [0, 2), \ g_6(t) = 1, \ t \in [2, 10],$$

with initial conditions

$$h_1(0) = h_2(0) = h_3(0) = h_6(0) = h_7(0) = 1, \quad h_5(0) = h_8(0) = 2, \quad h_4(0) = 7.$$

For the third experiment,

$$g_1(t) = 0,\ t \in [0, 1),\ g_1(t) = 1,\ t \in [1, 10],$$

$$g_2(t) = 0,\ t \in [0, 5),\ g_2(t) = 1,\ t \in [5, 10], \qquad g_6(t) = 1,\ t \in [0, 10],$$

with initial conditions

$$h_1(0) = h_2(0) = h_7(0) = 1, \quad h_3(0) = h_4(0) = h_5(0) = h_8(0) = 2, \quad h_6(0) = 6.$$

Finally, for the fourth experiment

$$g_1(t) = 0.1,\ t \in [0, 10], \quad g_2(t) = 0,\ t \in [0, 3),\ g_2(t) = 1,\ t \in [3, 10], \quad g_6(t) = 0.8,\ t \in [0, 10],$$

with initial conditions

$$h_1(0) = h_2(0) = h_4(0) = h_8(0) = 1, \quad h_5(0) = h_7(0) = 2, \quad h_3(0) = 4, \quad h_6(0) = 6.$$

5.2.1 Simulated Data

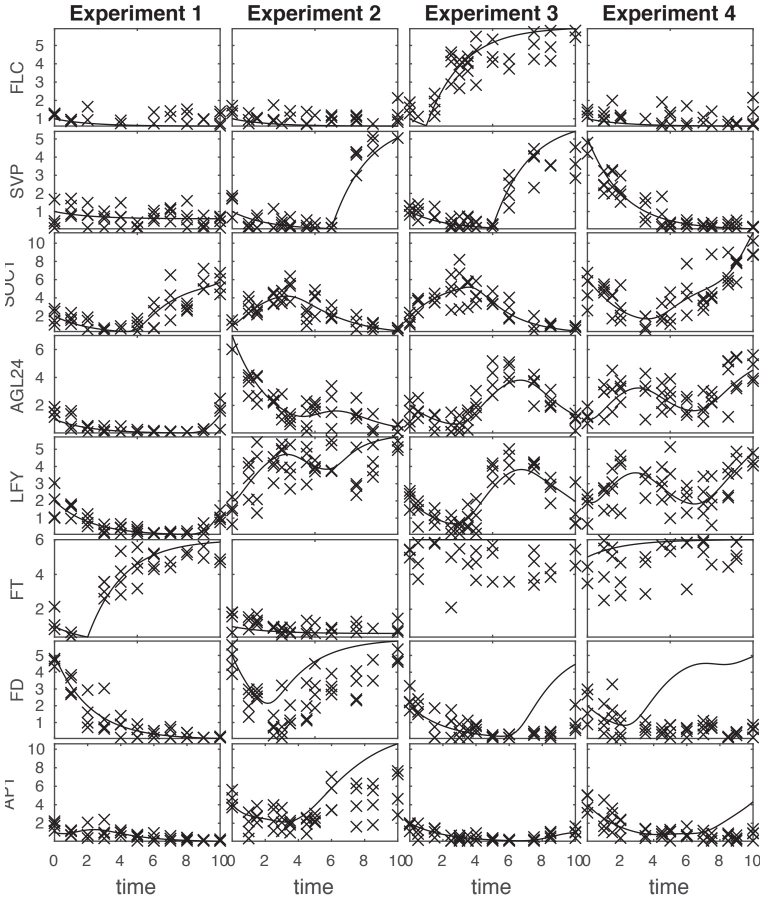

Figure 18: The pseudo-random data (crosses) generated for the simulation study of the flowering network of *A. thaliana* and their underlying deterministic profiles (solid lines).

5.2.2 Prior distributions

The prior distribution used for the results reported in Sect. **I**3.2. Note that the same hyperparameters are used for the repressed activation simulation study.

- for the degradation rate, δ, is a Gamma distribution with mean 0.3450 and standard deviation 0.1543,
- for the precision σ_k^{-2}, $k = 1, 2$, is a scaled χ^2 distribution with $n_{k,0} = 0.001$ degrees of freedom and scale $\sigma_{k,0}^2 = 0.001$
- for the number of regulators, a Poisson prior with parameter $\lambda = 0.15$
- for the choice of regulators, $\pi(f_i \in \varPhi) = 0.23, 0.18, 0.13, 0.03, 0.05, 0.20, 0.15, 0.02$ for $i = 1, \ldots, 8$, respectively.

- for the threshold priors the cut-off value $\bar{g}_{min}$ is set equal to the median of the standardized gradient values $\bar{g}_f^{(k)}(t)$ across the observed time-intervals of all experiments.

The prior distributions for the number of regulators, for the choice of regulators and for the threshold priors used in the results discussed in Sect. **I**3.2 are presented in Fig. S19.

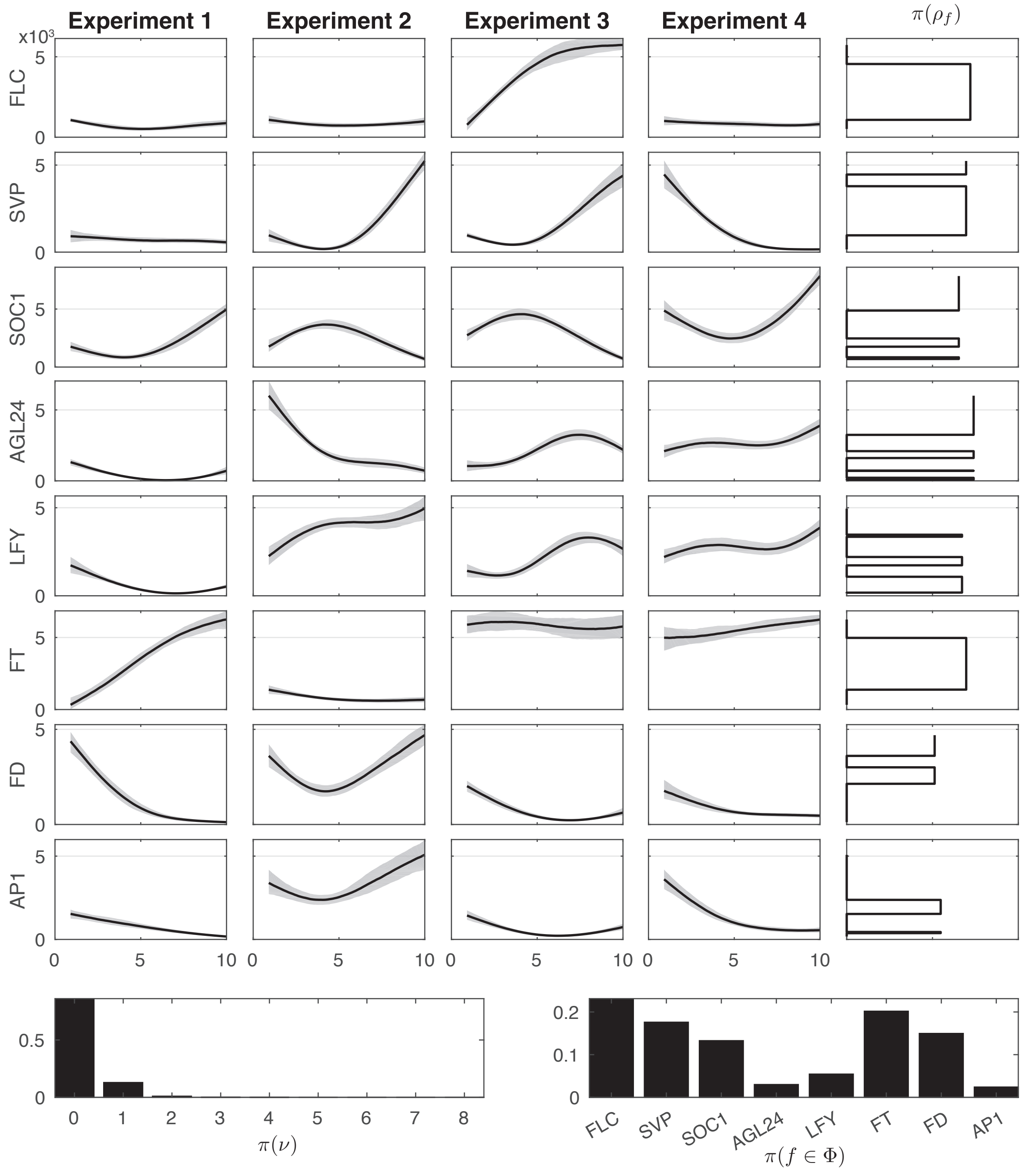

Figure 19: **Prior distributions for the simulation study of the flowering network of** ***A. thaliana*****.** The setup of the figure is the same as in Fig. S6 adapted to the larger number of candidate regulators and experimental conditions.

5.2.3 Monte Carlo Markov Chains of various parameters

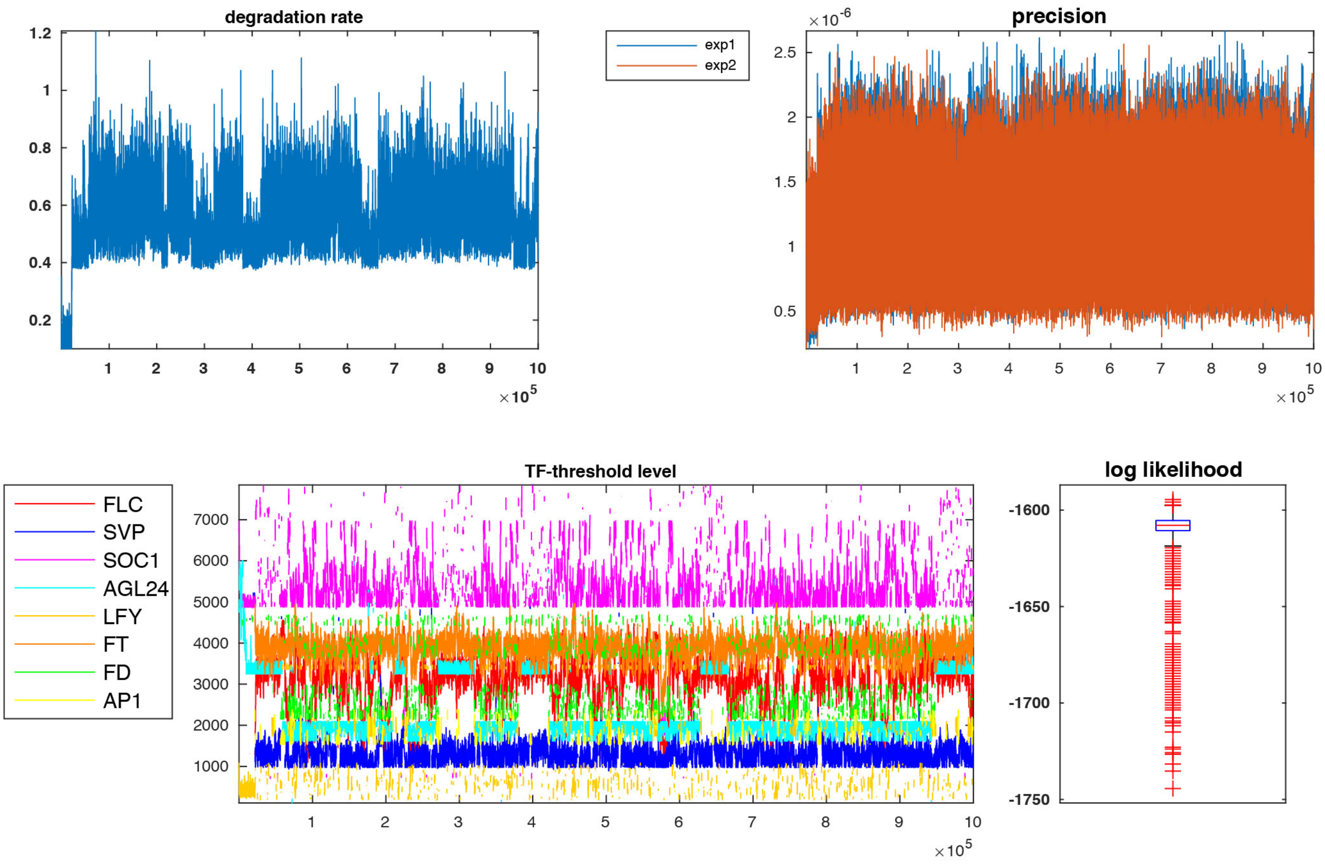

Figure 20: Monte Carlo Markov Chains of the parameters degradation rate (δ), precision (σ_k^{-2}) and threshold level (ρ_f, $f = f_1, f_2, \ldots, f_6, T$) of the TRS model for the flowering time simulation study discussed in Sect. **I**3.2.

Fig. 21 provides a summary of the results discussed in Sect. **I**3.2

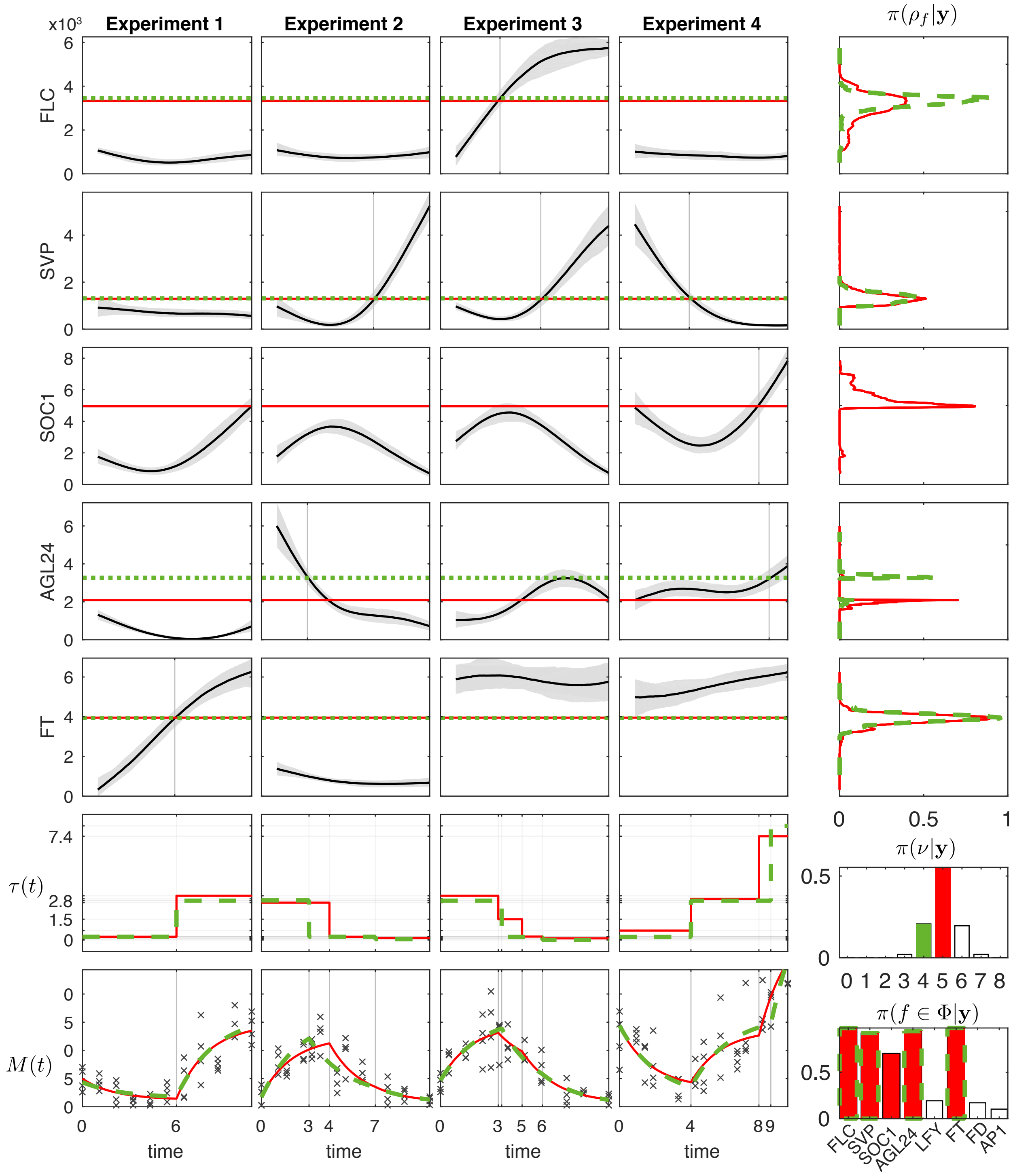

Figure 21: **Posterior inference for the simulation study of the repressed activation network using the TRS model.** This is a summary figure for the results discussed in Sect. I3.2. The setup of the figure is the same as Fig. S9.

5.2.4 Output of the GRNInfer tool

Table 4 provides the output of the GRNInfer tool.

	FLC	SVP	SOC1	AGL24	LFY	FT	FD	AP1
FLC	-0.5996	0.1066	0.0717	-0.0777	0.1549	0.0864	0.2087	-0.0811
SVP	0.2125	-0.3033	-0.0258	0.1110	0.0427	-0.2033	0.1901	0.1167
SOC1	0.0192	-0.4205	-0.8007	-0.1388	0.2035	0.0840	1.5930	-0.4279
AGL24	0.3867	-0.2753	0.1310	-0.7953	0.2178	-0.0683	0.1981	0.0150
LFY	0.0376	-0.0745	0.0779	0.5263	-0.8195	-0.0229	-0.1671	0.1959
FT	0.4417	0.0956	0.1636	0.1213	0.0065	-1.1441	1.3322	-0.2359
FD	0.6391	0.0070	-0.0406	-0.0397	0.0745	0.0039	-1.0987	0.3257
AP1	0.7293	0.2180	-0.0289	0.0713	0.1264	-0.2499	0.2343	-1.0412

Table 4: The output of the GRNInfer tool for the data produced in this simulation study. The (i, j) entry of the table corresponds to the coefficient of the regulation of the i-th gene by the j-th gene where the names of the regulating and target genes are given in the first row and first column, respectively.

6 Application to *A. Thaliana*

6.1 Target and regulator gene names

The first target gene has common name ANAC092 with the GST id number CATMA5a35200 and name AT5G39610. The following are the candidate regulator genes.

GST id	Name	Common name
AT1G03800	CATMA1a02660	ERF10
AT1G06160	CATMA1a05200	ORA59
AT1G25550	CATMA1a24250	AT1G25550
AT1G35560	CATMA1a33770	TCP23
AT1G61110	CATMA1a50150	ANAC025
AT3G02150	CATMA3a01150	TCP13
AT3G15510	CATMA3a14920	ANAC056
AT3G19290	CATMA3a18940	ABF4
AT3G23220	CATMA3a23220	ERF095(ESE1)
AT3G50260	CATMA3a43300	CEJ1
AT3G56530	CATMA3a49500	ANAC064
AT3G57600	CATMA3a50610	DREB2F
AT4G34000	CATMA4a35825	ABF3
AT4G34590	CATMA4a36430	GBF6
AT5G08330	CATMA5a07600	TCP21
AT5G13330	CATMA5a11530	Rap2.6L
AT5G53980	CATMA5a49890	ATHB52
AT5G58900	CATMA5a54650	AT5G58900
AT5G61270	CATMA5c65068	PIF7
AT5G62000	CATMA5a57616	ARF2

The second target gene has common name SCL3. The GST id number is CATMA1a41480 and the gene name AT1G50420. The candidate regulator genes are

GST id	Name	Common name
CATMA1a02660	AT1G03800	ERF10
CATMA1a05200	AT1G06160	ORA59
CATMA1a33770	AT1G35560	TCP23
CATMA2a22760	AT2G24430	ANAC038
CATMA3a01150	AT3G02150	TCP13
CATMA3a05630	AT3G06490	MYB108
CATMA3a18940	AT3G19290	ABF4
CATMA3a23220	AT3G23220	ERF095(ESE1)
CATMA3a50610	AT3G57600	DREB2F
CATMA5a07600	AT5G08330	TCP21
CATMA5a11530	AT5G13330	Rap2.6L
CATMA5a50510	AT5G54680	ILR3
CATMA5c65068	AT5G61270	PIF7
CATMA5a57616	AT5G62000	ARF2
CATMA5a60750	AT5G65410	ATHB25

6.2 Prior distributions

The hyperparameters used in this example are the same as in the two simulation studies.

Target: ANAC092

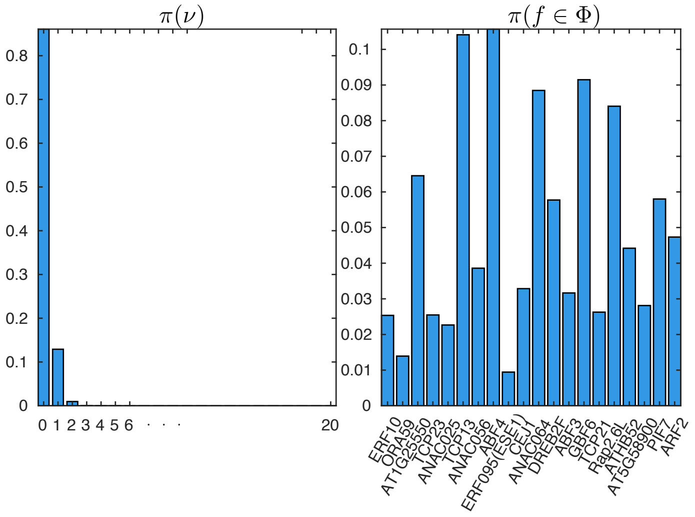

Figure 22: Prior distributions for the number (left) and choice of regulators (right) of the target ANAC092

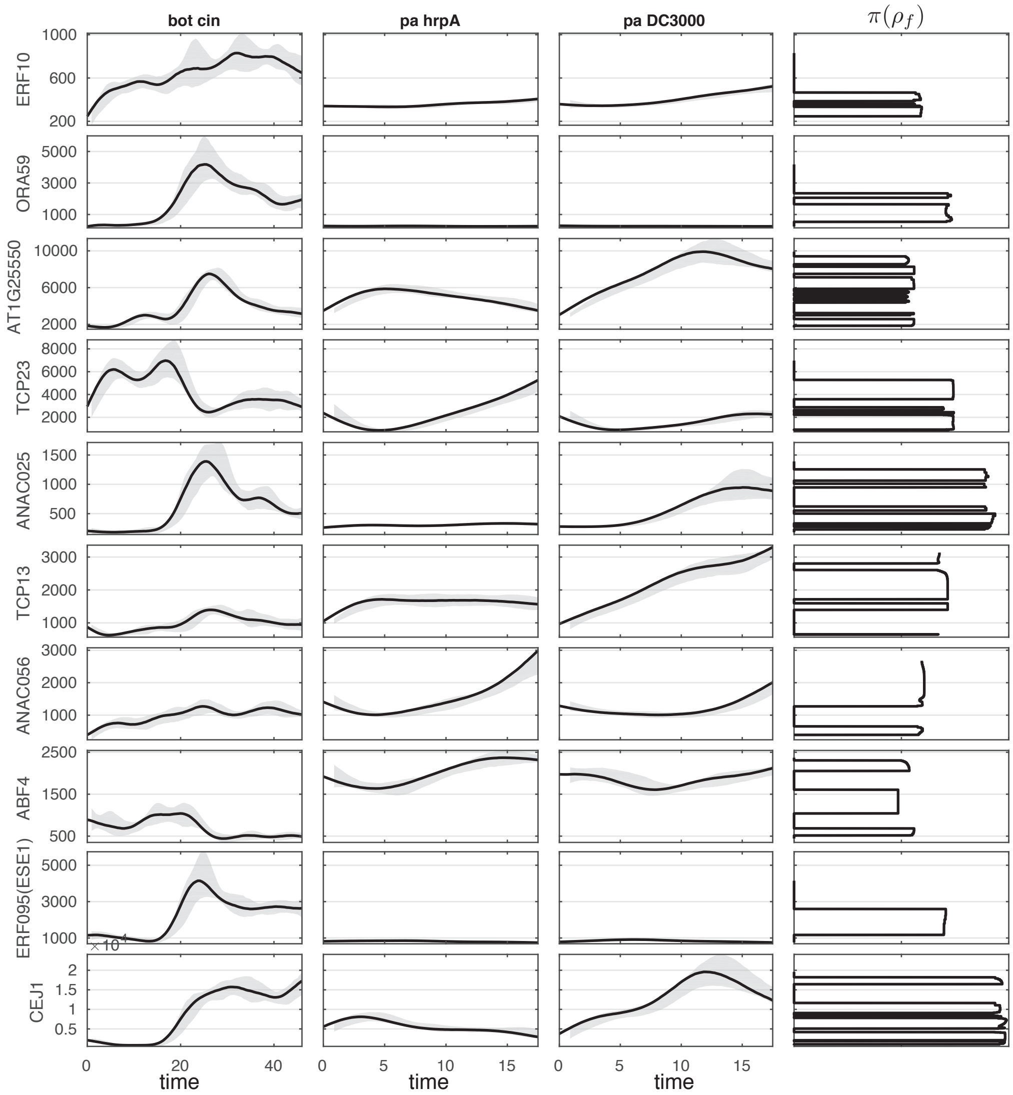

Figure 23: **Prior densities for the thresholds of the first ten candidate regulators of ANAC092**. The smoothed regulators profile (solid line) and Bootstrap 95% confidence envelopes (grey areas) of the candidate regulators are displayed in the first three columns, while the priors for their thresholds, $\pi(\rho_f)$, are displayed are provided in the last column.

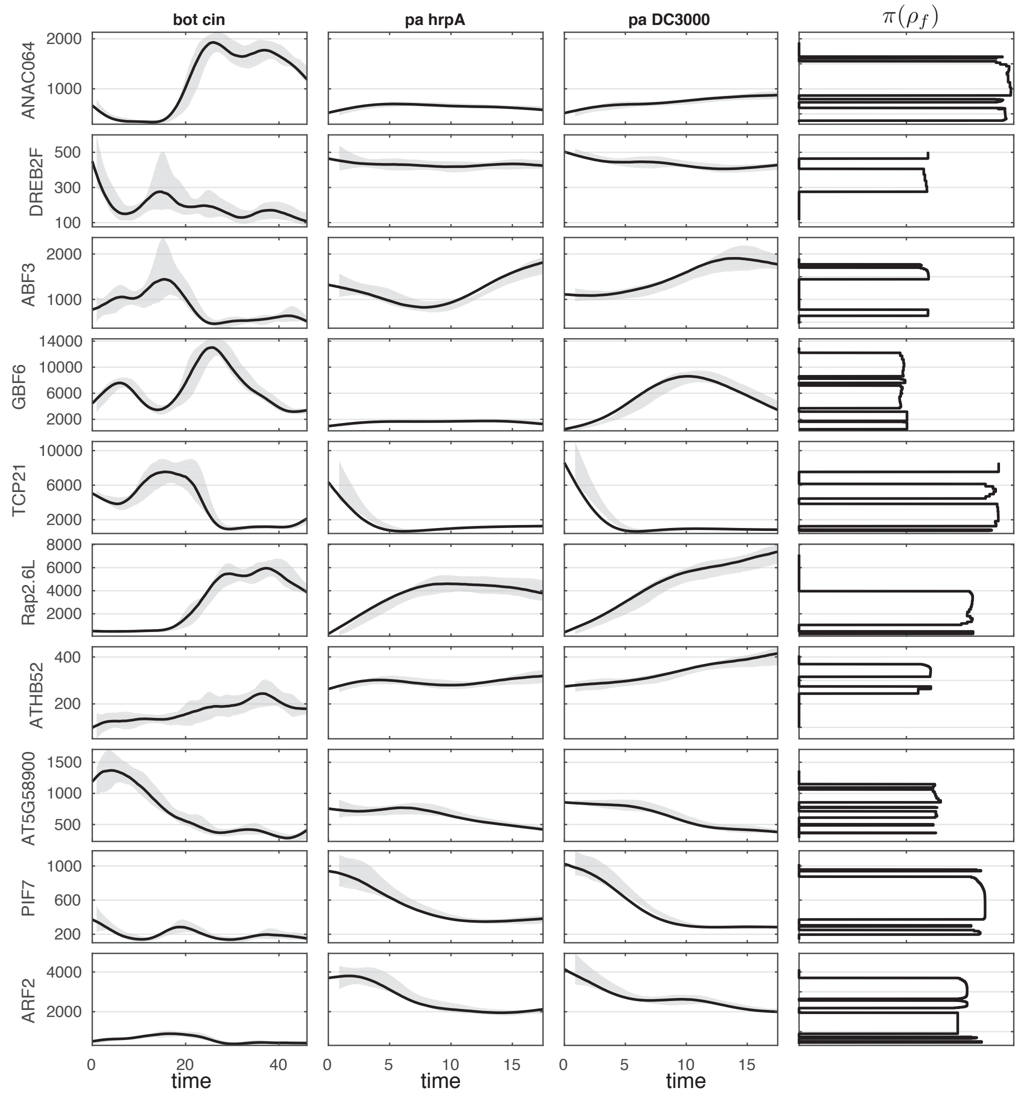

Figure 24: **Prior densities for the thresholds of the last ten candidate regulators of ANAC092**. The smoothed regulators profile (solid line) and Bootstrap 95% confidence envelopes (grey areas) of the candidate regulators are displayed in the first three columns, while the priors for their thresholds, $\pi(\rho_f)$, are displayed are provided in the last column.

Target: SCL3 Similarly, with the application related to the regulation of the target gene ANAC092, the hyperparameters used in this example are also the same as in the two simulation studies.

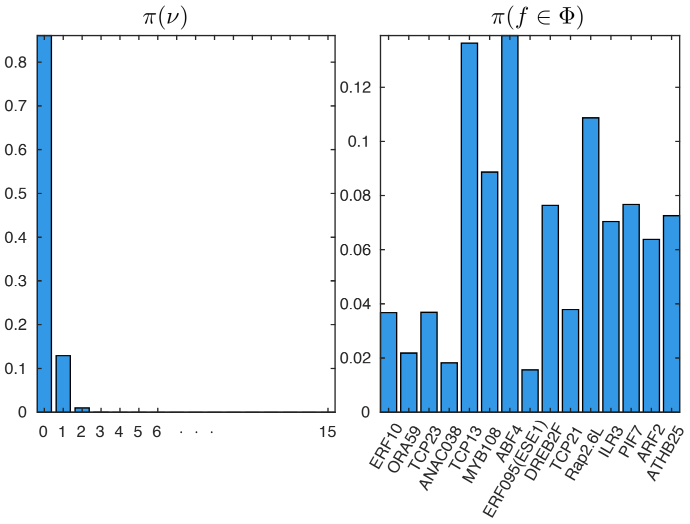

Figure 25: Prior distributions for the number (left) and choice of regulators (right) of the target SCL3

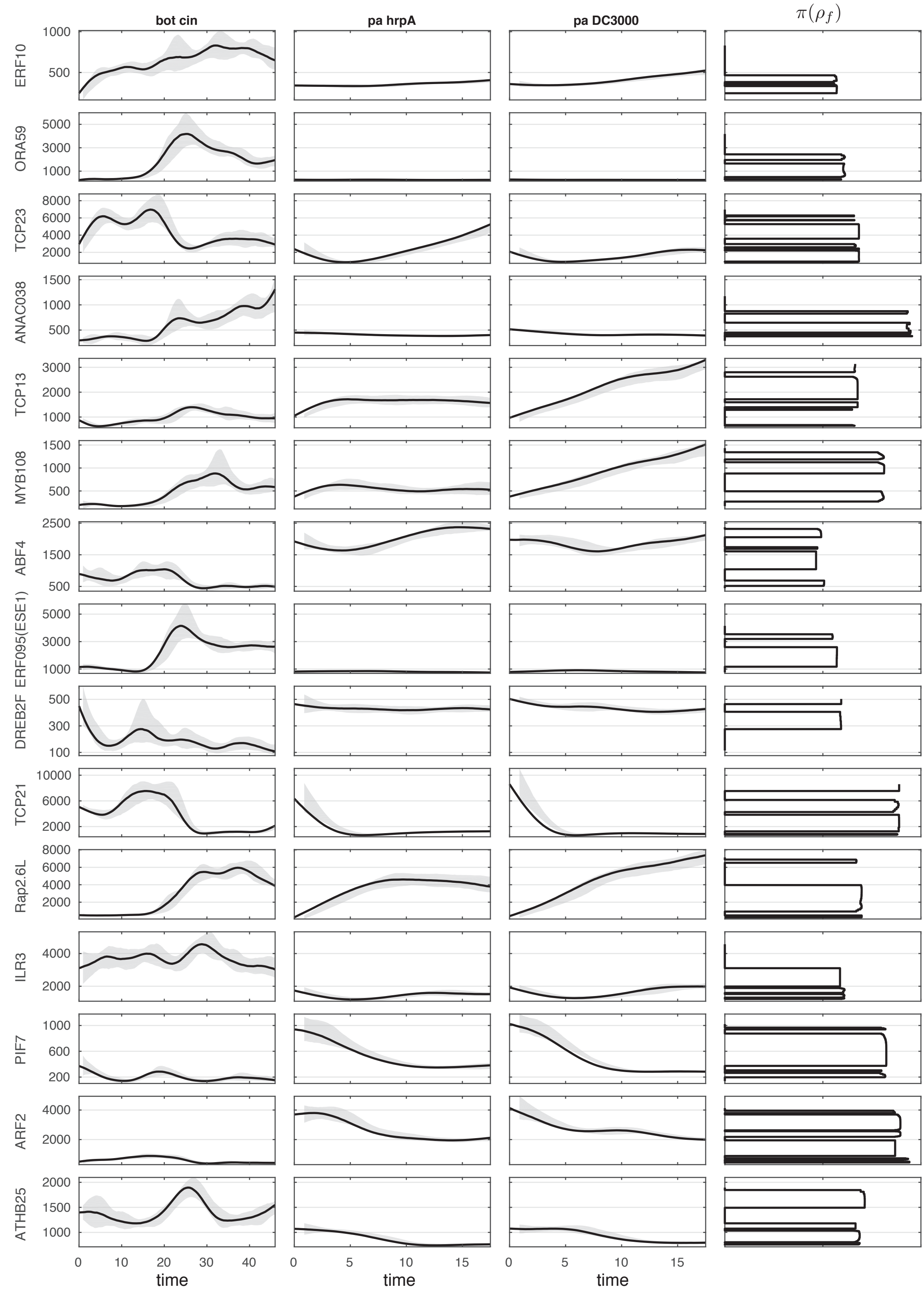

Figure 26: **Prior densities for the thresholds of the candidate regulators of SCL3**. The smoothed regulators profile (solid line) and Bootstrap 95% confidence envelopes (grey areas) of the candidate regulators are displayed in the first three columns while the priors for their thresholds, $\pi(\rho_f)$, are displayed are provided in the last column.

6.3 Monte Carlo Markov Chains of various parameters

Target: ANAC092

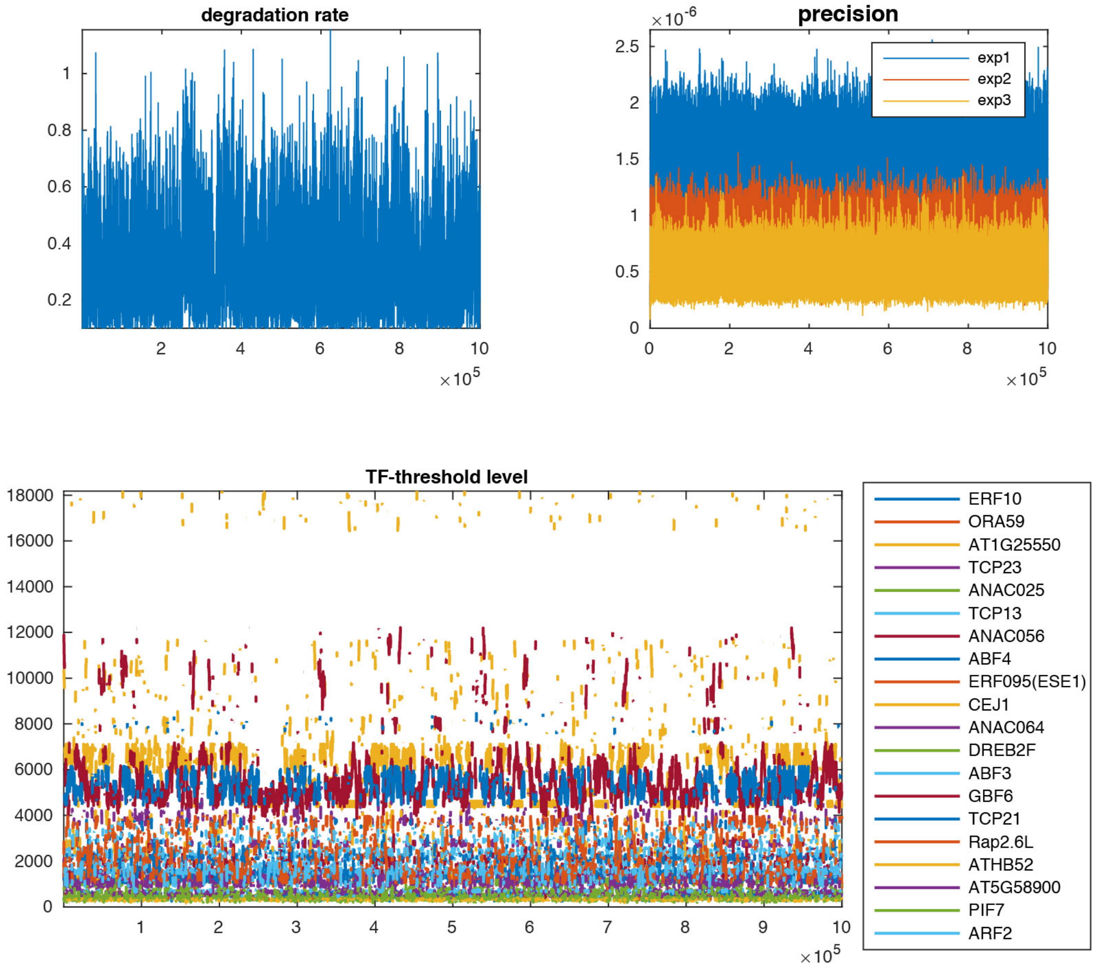

Figure 27: Monte Carlo Markov Chains of the parameters of the TRS model for the target gene ANAC092.

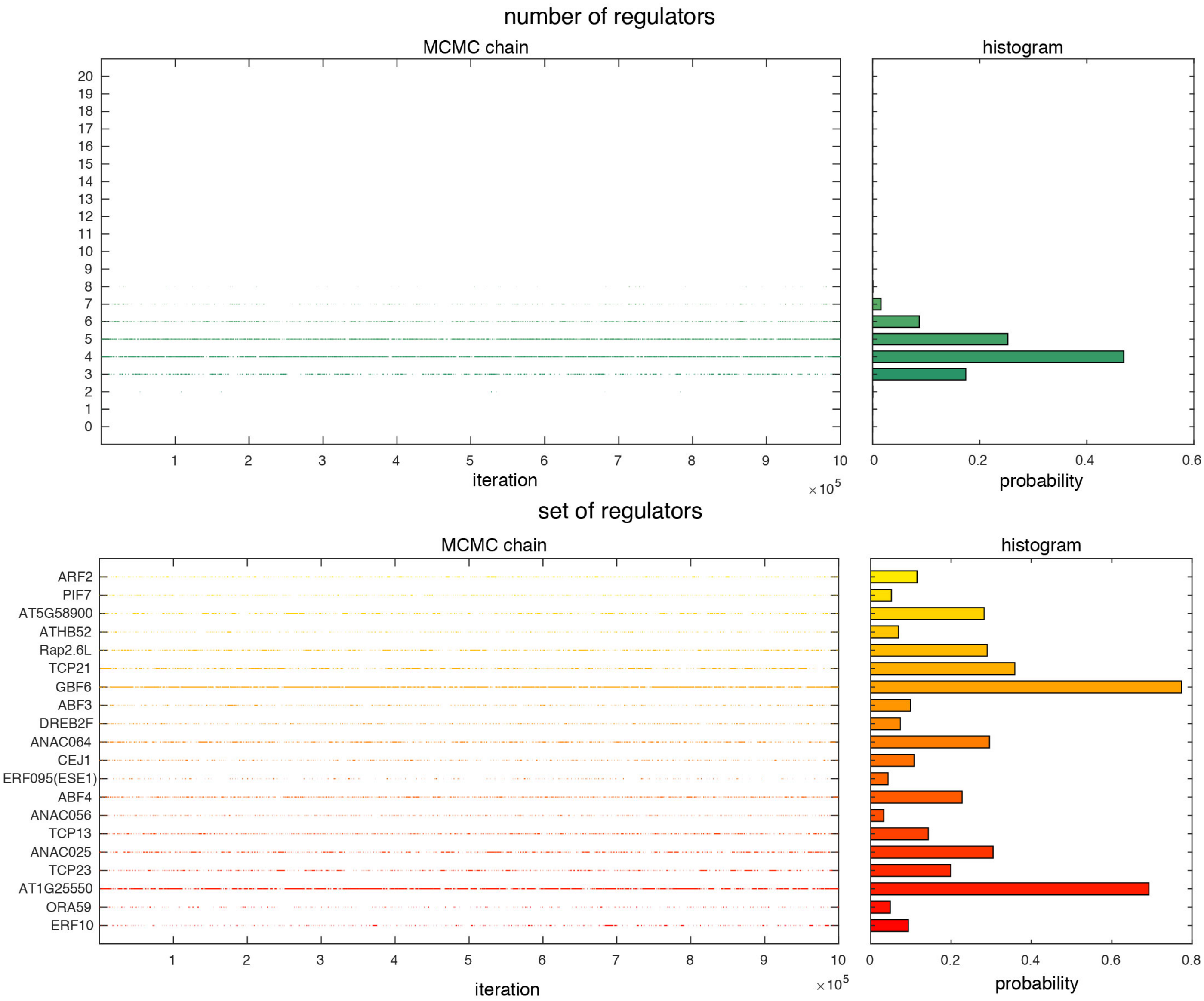

Figure 28: Monte Carlo Markov Chains of the number and choice of regulator of the TRS model for the target gene SCL3.

Target: SCL3

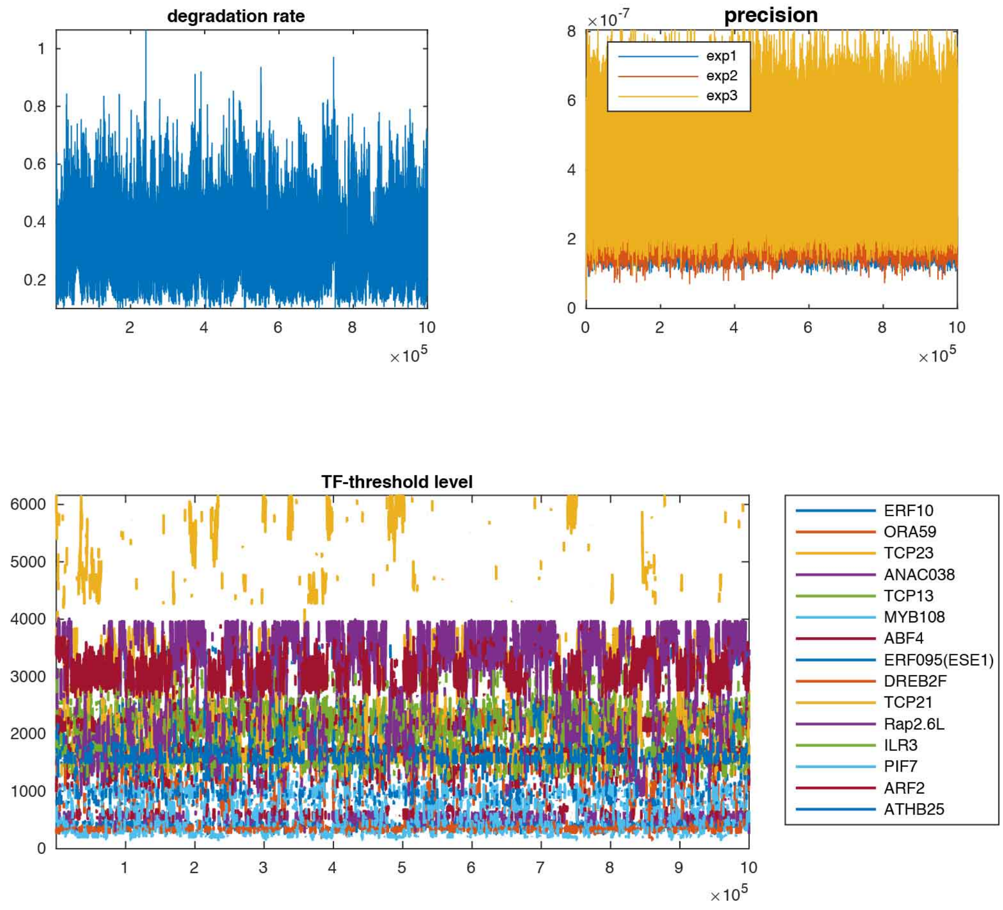

Figure 29: Monte Carlo Markov Chains of the parameters degradation rate (δ), precision (σ_k^{-2}) and threshold level (ρ_{f_j}, $j = 1, 2, \ldots, 15$) of the TRS model for the target gene SCL3.

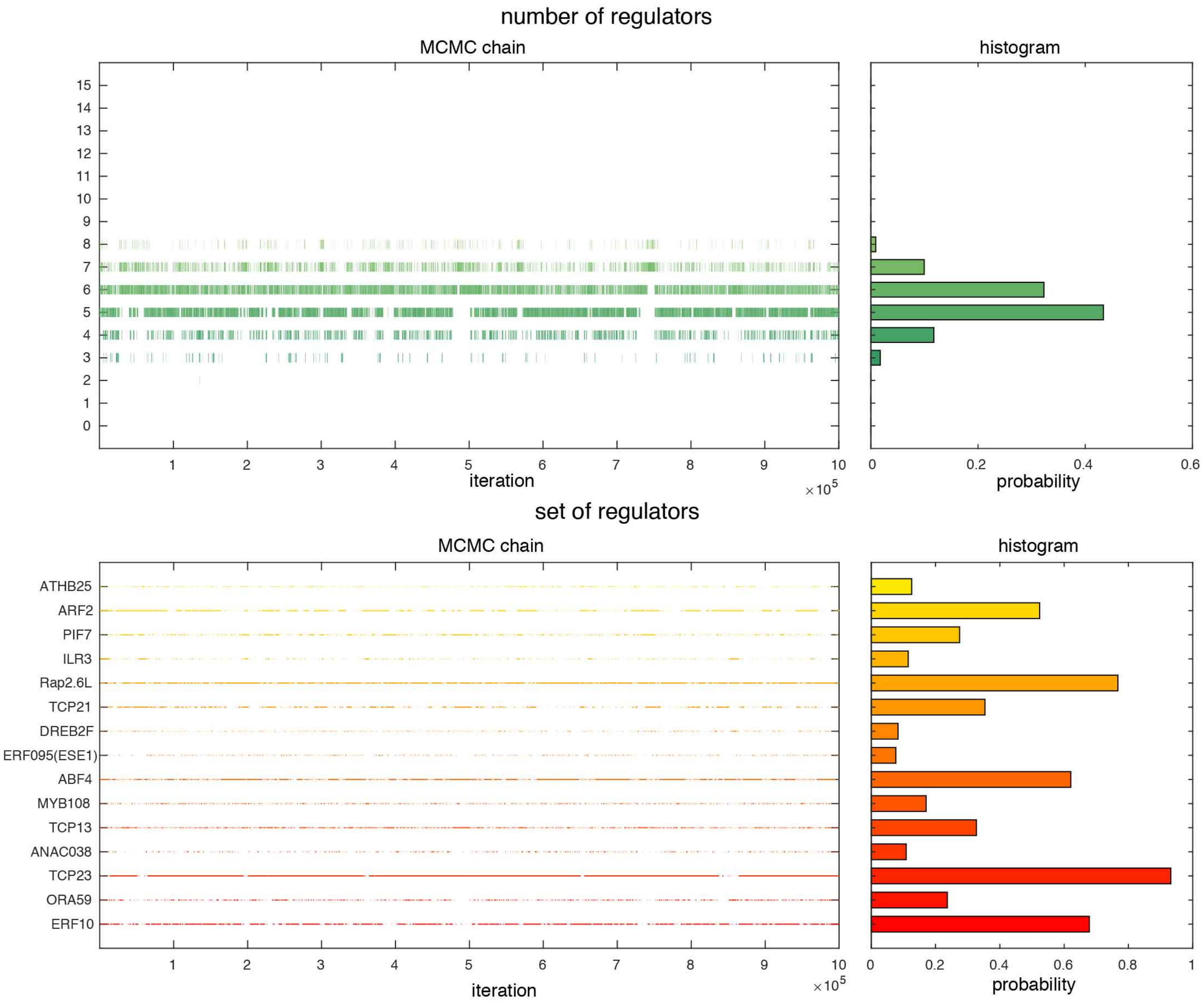

Figure 30: Monte Carlo Markov Chains of the number and choice of regulator of the TRS model for the target gene SCL3.

References

Bhaskaran, S., P., U., and Nair, A. S. (2015). *Hill Equation in Modeling Transcriptional Regulation*, pages 77–92. Springer Netherlands, Dordrecht.

Bornholdt, S. (2008). Boolean network models of cellular regulation: prospects and limitations. *Journal of The Royal Society Interface*, 5(Suppl 1):S85—-S94.

Han, S., Wong, R. K. W., Lee, T. C. M., Shen, L., Li, S.-Y. R., and Fan, X. (2014). A Full Bayesian Approach for Boolean Genetic Network Inference. *PLoS ONE*, 9(12):e115806.

Huynh-Thu, V. A., Irrthum, A., Wehenkel, L., and Geurts, P. (2010). Inferring Regulatory Networks from Expression Data Using Tree-Based Methods. *PLOS ONE*, 5(9):e12776.

Leal Valentim, F., van Mourik, S., Posé, D., Kim, M. C., Schmid, M., van Ham, R. C. H. J., Busscher, M., Sanchez-Perez, G. F., Molenaar, J., Angenent, G. C., Immink, R. G. H., and van Dijk, A. D. J. (2015). A Quantitative and Dynamic Model of the Arabidopsis Flowering Time Gene Regulatory Network. *PLOS ONE*, 10(2):e0116973.

Madar, A., Greenfield, A., Vanden-Eijnden, E., and Bonneau, R. (2010). DREAM3: network inference using dynamic context likelihood of relatedness and the inferelator. *PLoS One*, 5.

Ou-Yang, L., Yan, H., and Zhang, X.-F. (2017). Identifying differential networks based on multi-platform gene expression data. *Molecular BioSystems*, 13(1):183–192.

Santillán, M. (2008). On the Use of the Hill Functions in Mathematical Models of Gene Regulatory Networks. *Mathematical Modelling of Natural Phenomena*, 3(2):85–97.

Wang, Y., Joshi, T., Zhang, X.-S., Xu, D., and Chen, L. (2006). Inferring gene regulatory networks from multiple microarray datasets. *Bioinformatics*, 22(19):2413–2420.

Wu, C. F. J. (1986). Jackknife, Bootstrap and Other Resampling Methods in Regression Analysis. *Ann. Stat.*, 14(4):1261–1295.

Yip, K. Y., Alexander, R. P., Yan, K.-K., and Gerstein, M. (2010). Improved Reconstruction of In Silico Gene Regulatory Networks by Integrating Knockout and Perturbation Data. *PLOS ONE*, 5(1):e8121.
